# PACAP38/mast-cell-specific receptor axis mediates repetitive stress-induced headache in mice

**DOI:** 10.1186/s10194-024-01786-3

**Published:** 2024-05-28

**Authors:** Hyeonwi Son, Yan Zhang, John Shannonhouse, Ruben Gomez, Yu Shin Kim

**Affiliations:** 1 Department of Oral & Maxillofacial Surgery, School of Dentistry, University of Texas Health Science Center, San Antonio, TX USA; 2 Programs in Integrated Biomedical Sciences, Biomedical Engineering, Radiological Sciences, Translational Sciences, University of Texas Health Science Center, San Antonio, TX USA

**Keywords:** Migraine, Headache, PACAP38, MrgprB2, Stress, Mast cell, Peripheral sensitization, Trigeminal ganglion

## Abstract

**Background:**

Pain, an evolutionarily conserved warning system, lets us recognize threats and motivates us to adapt to those threats. Headache pain from migraine affects approximately 15% of the global population. However, the identity of any putative threat that migraine or headache warns us to avoid is unknown because migraine pathogenesis is poorly understood. Here, we show that a stress-induced increase in pituitary adenylate cyclase-activating polypeptide-38 (PACAP38), known as an initiator of allosteric load inducing unbalanced homeostasis, causes headache-like behaviour in male mice via mas-related G protein-coupled receptor B2 (MrgprB2) in mast cells.

**Methods:**

The repetitive stress model and dural injection of PACAP38 were performed to induce headache behaviours. We assessed headache behaviours using the facial von Frey test and the grimace scale in wild-type and MrgprB2-deficient mice. We further examined the activities of trigeminal ganglion neurons using in vivo *Pirt*-GCaMP Ca^2+^ imaging of intact trigeminal ganglion (TG).

**Results:**

Repetitive stress and dural injection of PACAP38 induced MrgprB2-dependent headache behaviours. Blood levels of PACAP38 were increased after repetitive stress. PACAP38/MrgprB2-induced mast cell degranulation sensitizes the trigeminovascular system in dura mater. Moreover, using in vivo intact TG *Pirt*-GCaMP Ca^2+^ imaging, we show that stress or/and elevation of PACAP38 sensitized the TG neurons via MrgprB2. MrgprB2-deficient mice showed no sensitization of TG neurons or mast cell activation. We found that repetitive stress and dural injection of PACAP38 induced headache behaviour through TNF-a and TRPV1 pathways.

**Conclusions:**

Our findings highlight the PACAP38-MrgprB2 pathway as a new target for the treatment of stress-related migraine headache. Furthermore, our results pertaining to stress interoception via the MrgprB2/PACAP38 axis suggests that migraine headache warns us of stress-induced homeostatic imbalance.

**Supplementary Information:**

The online version contains supplementary material available at 10.1186/s10194-024-01786-3.

## Background

Migraine headache is the leading cause of disrupted quality of life and is a major global health problem [1, 2]. There is a need for more effective treatments, although some treatments such as triptans, ergot derivatives, opioids, and barbiturates exist [3]. Migraine treatments have been recently developed through the efforts of many researchers, but we still don’t completely understand the “why and how” of migraine occurrence. Pituitary adenylate cyclase-activating polypeptide-38 (PACAP38), an endogenous neuropeptide, is a promising target for treating migraine headache because increased PACAP38 levels are closely related to the onset of migraine [4–7]. Interestingly, PACAP38 plays a key role in regulating stress responses leading to homeostasis imbalances that may initiate migraine [8]. These features suggest that PACAP38 mediates stress and the development of migraine headache, although the mechanism by which PACAP38 causes headache remains unknown.

PACAP38 transduces signals through canonical receptors, including pituitary adenylate cyclase-activating polypeptide type 1 receptor (PAC1), vasoactive intestinal peptide receptor 1 (VPAC1), and vasoactive intestinal peptide receptor 2 (VPAC2). It is tentatively thought that these receptors contribute to the development migraine, but the evidence is inconclusive [9]. It has also been suggested that PACAP38 does not directly contribute to activation of trigeminal ganglion (TG) neurons via conventional receptors such as PAC1, VPAC1, and VPAC2, whereas it is presumed that PACAP38 signals via orphan mas-related G protein-coupled receptor B3 (MrgprB3) (rat) [10–12]. PACAP38 binds to MrgprB3 [13–15] and it may bind to MrgprX2, the human orthologue of mouse MrgprB2, resulting in mast cell degranulation. Mast cell degranulation in dura mater induces the sensitization of TG neurons, which is a vital process in the initiation of headache [16–19]. Moreover, recently it has been reported in a phase 2 trial that monoclonal antibody against PACAP38 exhibits significant effectiveness in migraine patients [9]. Here, we sought to determine whether migraine headache is mediated by the PACAP38-MrgprB2 pathway in dura mater, which if correct, would provide a novel target for treating migraine headache and may provide insights into why primary headache, specifically migraine headache, occurs.

## Materials and methods

### Animals

*Pirt*-GCaMP3 mice were generated as described previously [20, 21]. In *Pirt*-GCaMP3 mice, GCaMP3 expression is driven by the *Pirt* promoter, which is a pan-DRG and TG-selective promoter, resulting in almost all primary sensory neurons expressing GCaMP3. MrgprB2 knock-out (KO) mice were purchased from The Centre for Phenogenomics (Ontario, Canada) and were backcrossed to C57BL/6 or *Pirt*-GCaMP3 mice. To generate PAC1 conditional KO mice, we first crossed C57BL/6N-*A*^*tm1Brd*^*Adcyap1r1*^*tm1a(KOMP)Wtsi*^/MbpMmucd mice (MMRRC #46,500) with B6N(B6J)-Tg(CAG-Flpo)1Afst/Mmucd mice (MMRRC #36,512). The IRES: lacZ trapping cassette was excised using methods described in previous studies [22, 23]. The resultant *PAC1* floxed/floxed mice were subsequently bred with *SM2*-cre-ERT2 (JAX# 019079) or *Pirt*-cre mice. Mice were housed three to five per cage with a 12-hour light/dark cycle and fed water and mouse chow ad libitum. Male mice used for experiments were 6–12 weeks old. All animal procedures were approved by the University of Texas Health at San Antonio (UTHSA) Animal Care and Use Committee (IACUC). All experiments were performed following the National Institute of Health Guide for the Care and Use of Laboratory Animals.

### Peptides and drugs

PACAP38 was purchased from Phoenix Pharmaceuticals (Burlingame, CA, USA). QWF, interleukin 6 (IL-6), R-7050, SB366791, and PA-8 were purchased from Tocris Bioscience/Bio-Techne Corporation (Minneapolis, MN, USA). Compound 48/80 was obtained from MilliporeSigma (St. Louis, MO, USA), and a stock solution was prepared by dissolving in distilled water. PACAP38 (1 ng/5 µL) and IL-6 (0.1 ng/5 µL) were prepared in synthetic interstitial fluid (SIF) [24] and administered to dura. R-7050 (10 mg/kg), SB366791 (1 mg/kg), and PA-8 (10 mg/kg) were prepared in 5% DMSO and 5% Tween-80 in 0.9% NaCl and administered intraperitoneally (i.p.).

### Repetitive restraint stress

Restraint stress was performed as previously described [25, 26] with modification. Animals were placed in tubular type restrainers (RWD, China) and were restrained for 1 h per day for 3 consecutive days. The restraint was carefully adjusted to permit sufficient respiration, and precautions were taken to prevent injury from the confinement restrainer. Control mice were isolated in a different room where they were subjected to a 1 h deprivation of food and water over three consecutive days. To prevent the potential transmission of stress characteristics, mice exposed to stress were housed distinctly apart from the control group.

### Behavioural tests

**The facial mechanical von Frey test** was performed according to previously published methods with some modifications [27]. Mice were habituated to the experimenter’s smell and hand touch for 2 days and acclimated in a plexiglass chamber with 4 oz paper cups for 2 h/d for 3 days. Baseline testing of cutaneous facial (periorbital region) sensitivity touched by von Frey filament was conducted for 3 days. The baseline of the facial von Frey test was set when a withdrawal threshold of approximately 0.5 to 0.7 g was reached. The thresholds were determined using the Dixon “up-and-down” method [28]. **Grimace pain behaviour** was measured in five characterized facial areas (orbital, nose, cheek, ears, and whiskers) on a scale of 0 to 2 (0 = not present, 1 = somewhat present, 2 = clearly present) as previously published [29]. We assessed orbital tightening, nose bulge, cheek bulge, ear position, and whisker change. The experimenter handling the mice and the data analyst were blinded to experimental conditions.

### TG exposure surgery for in vivo intact *Pirt*-GCaMP3 Ca^2+^ imaging

Mice were anesthetized by i.p. injection of Ketamine/Xylazine (approximately 80/10 mg/kg) (MilliporeSigma, St Louis, MO, USA), and ophthalmic ointment (Lacri-lube; Allergen Pharmaceuticals) was applied to the eyes. The right-side dorsolateral skull was exposed by removing skin and muscle. A patch of dorsolateral skull (parietal bone between the right eye and ear) was removed using a dental drill (Buffalo Dental Manufacturing, Syosset, NY, USA) to make a cranial window (∼ 10 × 10 mm). The TG was then exposed by aspirating overlying cortical tissue. During the surgery, the mouse’s body temperature was maintained on a heating pad at 37 °C ± 0.5 °C and monitored by rectal probe. We performed in vivo imaging one day after the cessation of repetitive stress for stressed mice, while PACAP38-injected mice were used 3 h after injection.

### In vivo intact TG *Pirt*-GCaMP3 Ca^2+^ imaging

In vivo intact TG *Pirt*-GCaMP3 Ca^2+^ imaging in live mice was performed for 2–5 h immediately after exposure surgery as previously described [20]. After the exposure surgery, mice were laid abdomen-down on a custom-designed platform under the microscope. For in vivo intact TG *Pirt*-GCaMP3 Ca^2+^ imaging, the animal’s head was fixed by a head holder to minimize movements from breathing and heartbeats. During the imaging session, body temperature was maintained at 37 °C ± 0.5 °C on a heating pad and monitored by rectal probe. Anaesthesia was maintained with 1–2% isoflurane using a gas vaporizer with pure oxygen. Live images were acquired at ten frames per cycle in frame-scan mode at ∼ 4.5 to 8.79 s/frame, ranging from 0 to 100 μm, using a 5 × 0.25 NA dry objective at 512 × 512 pixels or higher resolution with solid diode lasers tuned at 488 nm and emission at 500–550 nm. An average of 2,893 ± 59 neurons per TG (∼ 10% of total TG neurons [30, 31]) were imaged (∼ 2 Hz), and small regions of TG neurons were imaged at greater speed (> 40 Hz). von Frey filaments (0.4 g) and noxious water (4 °C and 50 °C) were applied to the different divisions of the animal’s face divided by TG branches for one imaging cycle after four baseline imaging cycles. Noxious hot water (2 mL) was gently applied with a pipette to the fur skin of the mouse’s face. Capsaicin (500 µM, 10 µl) was injected intracutaneously into the different TG branches using a syringe after four baseline imaging cycles. To prevent mechanical stimulation while injecting capsaicin into the skin, the syringe needle was inserted into the skin before imaging.

For imaging data analysis, raw image stacks were collected, deconvoluted, and imported into ImageJ (NIH). Optical planes from sequential time points were re-aligned and corrected using the stackreg rigid-body cross-correlation-based image alignment plugin. Ca^2+^ signal amplitudes were expressed as F_t_ (fluorescence intensity in each frame)/F_0_ (average fluorescence intensity during the first one to four frames). All responding cells were verified by visual examination of the raw imaging data. Neurons displaying changes in amplitude originating from Ca^2+^ signals without any external stimulation were categorized as spontaneous neurons. In contrast, neurons exhibiting changes in amplitude following specific stimuli were classified as activated neurons. We employed ImageJ software equipped with Feret’s diameter analysis tool to measure the diameter of the cell bodies of the collected cells. Based on this analysis, cells with a diameter of less than 20 μm were classified as small, those with a diameter between 20 and 25 μm were classified as medium, and those exceeding 25 μm were categorized as large diameter neurons.

### Dura injection

Dura injection was performed as previously described [27]. Drugs or compounds were injected into the junction of the sagittal and lambdoid sutures of dura mater in a volume of 5 µl via a modified internal cannula (P1 Technologies, Roanoke, VA, USA) under brief anaesthesia by isoflurane. The length of the injection needle was adjusted to 0.5 to 0.8 mm.

### In vivo imaging in dura mater

Mice were anesthetized by i.p. injection of Ketamine/Xylazine (80/10 mg/kg) (MilliporeSigma, St Louis, MO, USA), and ophthalmic ointment was applied to the eyes. The scalp was shaved and sterilized using 70% EtOH. To form a cranial window, a ∼ 3 × 3 mm round area of the right parietal skull was carefully removed. Using dental cement (Lang Dental Manufacturing, Wheeling, IL, USA), a crown was made around the cranial window, and the brain was covered with SIF. Texas-Red conjugated 2k Dalton dextran (Nanocs) was injected into the tail vein (100 µl of 1 mg/ml in saline) to visualize blood vessels in dura mater. Anesthetized mice were imaged with a single photon confocal microscope (Carl Zeiss) using a 40× water-immersion objective (1.0 NA, Carl Zeiss). The vessel diameter and Ca^2+^ transient of trigeminal afferent nerve fibres were measured using ImageJ software.

### Immunofluorescence imaging in dura mater

Animals were anesthetized with Ketamine/Xylazine (80/10 mg/kg) 3 h after PACAP38 injection (MilliporeSigma, St Louis, MO, USA), and cardiac perfusion fixation was conducted using 4% paraformaldehyde. After fixation, the dura mater was dissected and postfixed for 24 h in 4% paraformaldehyde. The collected tissues were incubated with primary antibodies: PACAP (Santa Cruz), βIII-tubulin (Abcam), and Rhodamine Avidin D (Vector Laboratories) for 24 h. Tissues were then incubated with Alexa-Fluor 488 or 647 (1:500; Invitrogen)-labelled polyclonal secondary antibodies, and slides were cover-slipped using a mounting medium (Invitrogen). Degranulated mast cells were identified by the presence of scattered granules adjacent to the cell.

### Mast cell culture and imaging

Peritoneal mast cells were isolated from adult male and female mice as previously described [32]. Briefly, 5 mL of ice-cold mast cell dissociation medium (MCDM; HBSS with 3% FBS and 10 mM HEPES, pH7.2) was injected into the peritoneal cavity, and the abdomen was massaged for 60s; peritoneal fluid was collected and centrifuged at 200 g for 5 min at room temperature. The pellets were resuspended in 2 mL MCDM, layered over 4 mL of isotonic 70% Percoll solution (MilliporeSigma, St Louis, MO, USA), and centrifuged at 500 g for 20 min at 4 °C. The purity of isolated mast cells was > 95%, as determined by avidin staining. The mast cells were resuspended in DMEM with 10% FBS, 100 U/mL penicillin, 50 mg/mL streptomycin, and 25 ng/mL recombinant mouse stem cell factor (mSCF; Peprotech, Cranbury, NJ, USA) and plated onto glass coverslips coated with 30 mg/mL fibronectin (MilliporeSigma, St Louis, MO, USA). LAD2 human mast cells (Laboratory of Allergic Diseases 2), provided by Professor Dr. Xinzhong Dong from Johns Hopkins University (MD, USA), were cultured in StemPro-34 SFM medium (Life Technologies, Carlsbad, CA, USA) supplemented with 2 mM L-glutamine, 100 U/ml penicillin, 50 mg/ml streptomycin, and 100 ng/ml recombinant human stem cell factor (Peprotech, Cranbury, NJ, USA) at 37 °C, 5% CO_2_. Cell culture medium was hemi-depleted every week and replaced with fresh medium. For Ca^2+^ imaging, the cell suspensions were seeded onto glass coverslips coated with fibronectin and incubated for 2 h (37 °C, 5% CO2). The cells were loaded with Fluo-8 Ca^2+^ dye (5 µM) for 30 min and imaged in Ca^2+^ imaging buffer (CIB; 125 mM NaCl, 3 mM KCl, 2.5 mM CaCl2, 0.6 mM MgCl2, 10 mM HEPES, 20 mM glucose, 1.2 mM NaHCO3, 20 mM sucrose, adjusted to pH 7.4 with NaOH) using a confocal (Carl Zeiss, Oberkochen, Germany) or widefield fluorescence microscope (Carl Zeiss) system.

### Ca^2+^ imaging in HEK293 cells

HEK293 cells were transiently transfected with lipofectamine 2000 (Invitrogen) using a total of 2 µg cDNA per dish (35 mm). Plasmid DNAs encoding the human MrgprX2 (pcDNA3.1) and mouse MrgprB2 (pcDNA3.1), provided by Professor Dr. Xinzhong Dong from Johns Hopkins University (MD, USA), were co-transfected with Gα15 (pcDNA3.1) at a ratio of 9:1. A surrogate expression marker, tdTomato (60 ng), was co-transfected along with other plasmids. Forty-eight hours following transfection, cells were loaded with AM esters of the Ca^2+^ indicators, Fluo-8 (1 µM; Abcam, Cambridge, MA, USA) along with Pluronic F-127 (0.04%; Life Technologies, Carlsbad, CA, USA) for 30–45 min at 37 °C in the dark, in standard extracellular solution (SES) containing 140 mM NaCl, 4 mM KCl, 2 mM CaCl2, 1 mM MgCl2, 10 mM HEPES, 10 mM D-glucose, pH 7.40. Cells were viewed on an upright Zeiss Examiner/A1 microscope fitted with a 40× water-immersion objective (0.75 NA, 2.1 mm free working distance, Carl Zeiss) and with an Axiocam 705 color camera (Carl Zeiss). Fluorescence images were taken every 5 s using the Zeiss Zen Blue software module. Cells were imaged in SES at room temperature; drugs were bath applied into the chamber at 400 µL/min following 10 cycles of baseline imaging, and responses were monitored for an additional 50 cycles. The percentage of cells responding to PACAP38 among total tdTomato-expressing cells was calculated to quantify the PACAP38 response.

### Mast cell degranulation assay

Mast cell degranulation was measured by β-hexosaminidase release assay as previously described for peritoneal mast cells [33] or LAD2 [34]. Isolated mouse mast cells or LAD2 were cultivated and then sensitized overnight with mouse monoclonal anti-DNP-IgE (MilliporeSigma) or human IgE (MilliporeSigma). Cells were washed three times and resuspended in HEPES buffer. The cells were incubated with PACAP38 (1 µM, Phoenix Pharmaceuticals) and Compound 48/80 (1 µg/ml, MilliporeSigma) for 45 min at 37 C°. β-hexosaminidase released into the supernatants and cell lysates was quantified by hydrolysis of p-nitrophenyl N-acetyl-β-D-glucosamide (MilliporeSigma) in 0.1 M sodium citrate buffer (pH 4.5) for 90 min at 37 °C. The percentage of β-hexosaminidase release was calculated as a percent of total content.

### ELISA

Dura mater and blood were collected between 8:00 and 10:00 a.m. after restraint stress. The collected dura maters were washed with PBS to remove blood. Blood was collected in vacutainers containing K3EDTA and centrifuged at 1000 g for 15 min at 4 °C. PACAP38 levels were determined using the PACAP38 ELISA kit (Antibodies.com, Cambridge, UK).

### Statistical analysis

All statistical analyses were performed in Prism (GraphPad). Error bars are defined as the mean ± S.E.M. Differences were considered significant at *p* < 0.05. Use of statistical tests are noted in the figure legends.

## Results

### PACAP38-MrgprB2 pathway mediates stress-induced headache behaviour

**Fig. 1:**
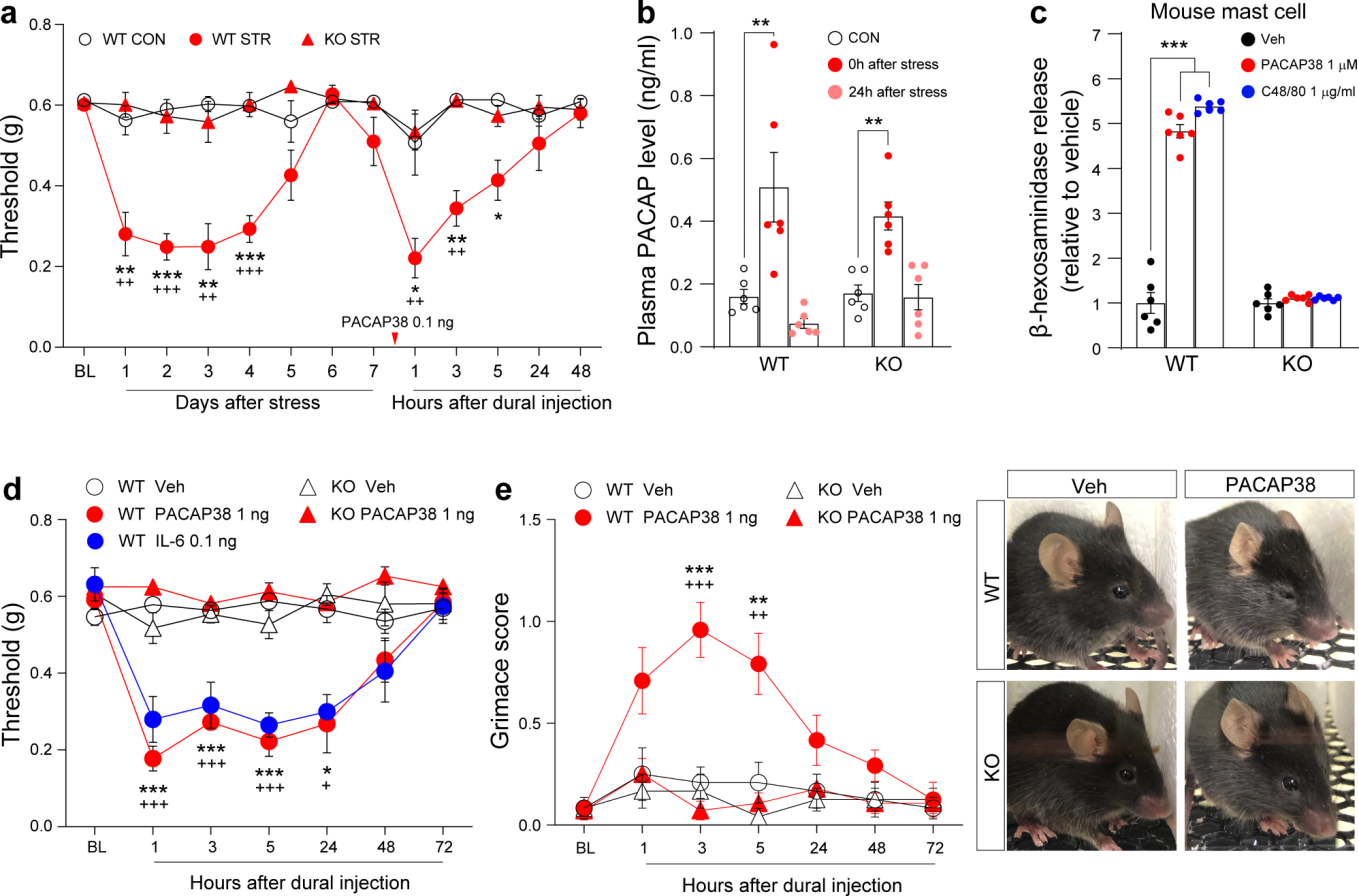
PACAP38-MrgprB2 pathway mediates stress-induced headache behaviours. **a** Facial mechanical withdrawal thresholds at consecutive time points after restraint stress (1 h/day for 3 days) and PACAP38 dural injection (0.1 ng) in wild-type (WT) or MrgprB2-deficient (KO) mice (*n* = 5 WT CON, 6 WT STR, and 9 KO STR mice). **b** Blood concentration of PACAP38 (*n* = 6 mice per group). Blood was collected immediately after cessation of stress or 24 h after stress. **c** β-hexosaminidase release by mouse peritoneal mast cells (*n* = 6 wells per group). **d** Facial mechanical withdrawal thresholds after dural injection with PACAP38 (1 ng) or IL-6 (0.1 ng) in WT or KO mice (*n* = 7 WT Veh, 7 WT PACAP, 5 WT IL-6, 6 KO Veh, and 6 KO PACAP mice). **e** Grimace score (*n* = 6 mice per group). (*right*) Representative mouse facial expression images. CON: control, C48/80: compound 48/80, STR: stress, IL-6: interleukin-6, Veh: vehicle. Significance comparisons: (a) *WT STR vs. WT CON; ^+^WT STR vs. KO STR; (d, e) *WT Veh. vs. WT PACAP38; ^+^WT PACAP38 vs. KO PACAP38. Error bars indicate S.E.M. *^/+^*p* < 0.05; **^/++^*p* < 0.01; ***^/+++^*p* < 0.001; (a, d, and e) two-way ANOVA with Tukey’s multiple comparison post-hoc test; (b and c) one-way ANOVA with Tukey’s multiple comparison post-hoc test

To determine whether MrgprB2 plays a role in stress-induced headache behaviour, we used a well-established stress model [25, 26]. Mice were exposed to restraint stress for 1 h/day for 3 days. The restrained mice showed hypersensitivity in the periorbital region for 5 days following restraint (Fig. 1a), similar to previous studies [25]. Hypersensitivity in the periorbital region was completely abolished by MrgprB2 deficiency as a result of KO of the MrgprB2 gene (Fig. 1a).

Because increased PACAP38 levels are observed during headache [4] and in patients with post-traumatic stress disorder [35], we investigated whether stress elevates PACAP38 levels in blood and dura mater. We collected blood and dura mater immediately after cessation of 1 h restraint stress and 24 h after cessation of stress. We found elevated PACAP38 levels in the blood immediately after stress but not at 24 h after stress in both wild-type (WT) and MrgprB2 deficient mice (Fig. 1b). There was no change of PACAP38 levels in dura mater (Supplementary Fig. 2a, see Additional file 1), which suggests that circulating PACAP38 contributes to the development of stress-induced headache behaviour rather than PACAP38 released from dura mater.

Stress induces mast cell degranulation in dura mater, which could be a cause of migraine headache [36, 37]. Consistent with this, we confirmed that PACAP38 application increased degranulation in peritoneal mast cells from WT mice but not in mast cells from MrgprB2-deficient mice (Fig. 1c). PACAP38-induced degranulation was also observed in the human mast cell line LAD2 (Supplementary Fig. 2b, see Additional file 1). These results point toward the possibility that PACAP38 mediates stress-induced mast cell degranulation resulting in headache behaviour. Therefore, to test whether elevated PACAP38 levels in dura mater induce headache behaviour, we directly injected PACAP38 into dura mater of WT and MrgprB2-deficient mice. Hypersensitivity in the periorbital region lasting 24 h was observed in WT PACAP38-injected mice (Fig. 1d). This tendency of hypersensitivity was similar to hypersensitivity observed previously in an IL-6-induced migraine model (Fig. 1d) [27]. Grimace score was also significantly increased in WT mice after PACAP38 injection and lasted 24 h (Fig. 1e). However, MrgprB2-deficient mice did not exhibit headache behaviours after PACAP38 injection (Fig. 1d and e). Injection of a 10-fold lower dose of PACAP38 into dura mater showed strong hypersensitivity in previously stressed WT but not in MrgprB2-deficient mice (Fig. 1a).

A recent study suggested that known PACAP38 receptors PAC1, VPAC1, and VPAC2 were not involved in the activation of TG neurons via PACAP38 [10], but some reports showed that PAC1 KO or blockade reduced the nociceptive response [38, 39]. We tested PAC1 conditional KO mice (*Pirt*-cre:*PAC*1 floxed/floxed for sensory neuron specific KO and *SM2*-cre:*PAC*1 floxed/floxed for blood vessel specific KO) for testing changes in headache behaviour. We did not see any reduced effect on headache behaviour in the conditional PAC1 KO mice (Supplementary Fig. 1a and b, see Additional file 1). In addition, the PAC1 inhibitor PA-8 did not block stress-induced headache behaviour (Supplementary Fig. 1c, see Additional file 1). Consistent with the effect of the PAC1 inhibitor in stressed mice (Supplementary Fig. 1c, see Additional file 1), the PAC1 inhibitor had no effect on PACAP38-induced headache behaviour (Supplementary Fig. 1d, see Additional file 1). These results indicate that PACAP elevation causes stress-induced headache behaviour through mast cell MrgprB2.

### PACAP38 directly activates MrgprB2

**Fig. 2:**
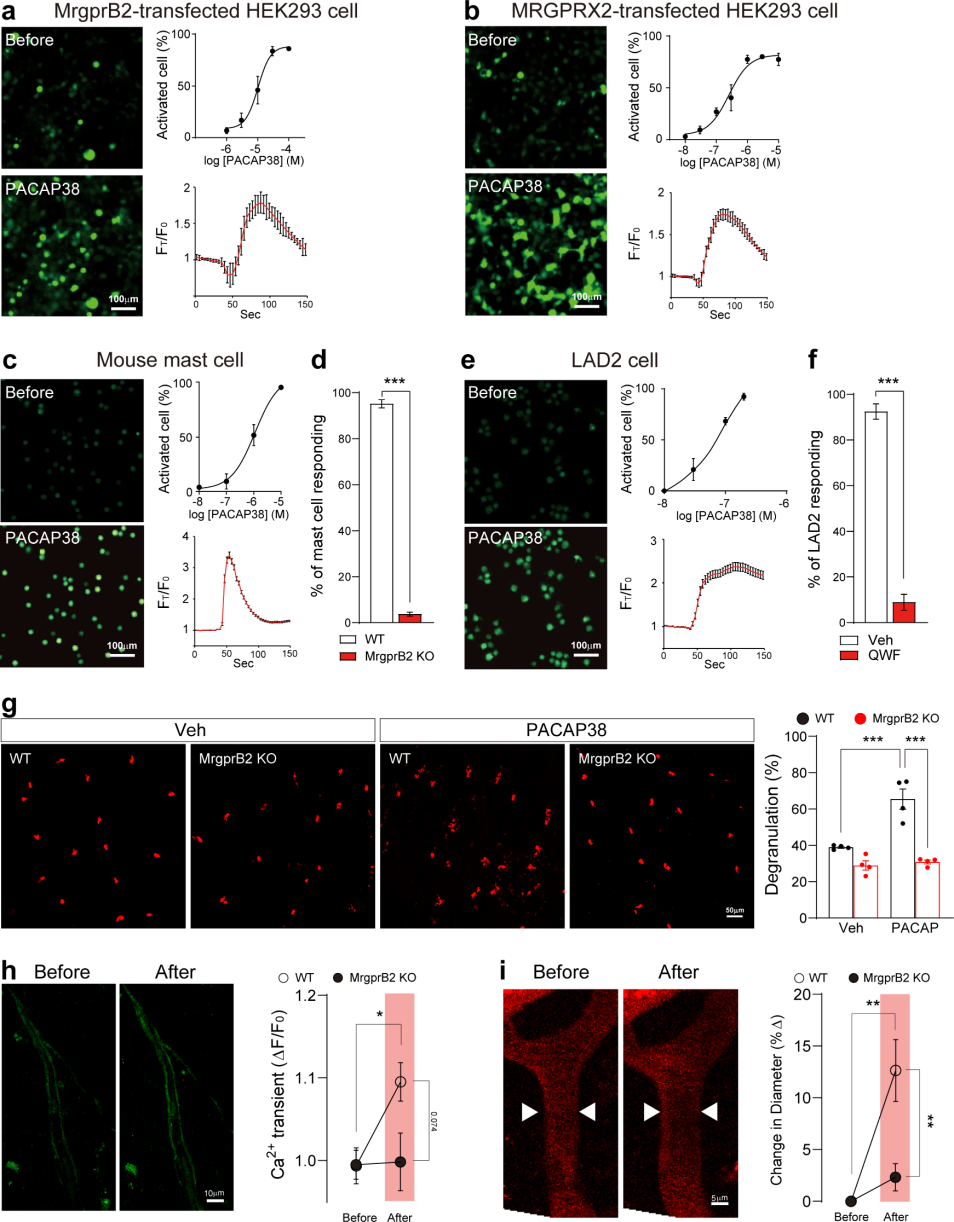
PACAP38 activates MrgprB2/MRGPRX2 and induces mast cell degranulation in dura mater for trigeminovascular alternations. **a and b** (*left*) Representative Ca^2+^ fluorescence images showing increase in HEK293 cells expressing MrgprB2 (a) or MRGPRX2 (b) with PACAP38 application (1 µM). (*right*, *top*) Percentage of cells activated by PACAP38 in MrgprB2-transfected cells (*n* = 13 cultures) (a) or MRGPRX2-transfected cells (*n* = 20 cultures) (b). (*right*, *bottom*) Average Ca^2+^ transient trace of cells activated by PACAP38 from MrgprB2 (10 µM)-transfected cells (a) or MRGPRX2 (1 µM)-transfected cells (b). **c and e** (*left*) Representative Ca^2+^ fluorescent images showing increase by PACAP38 (50 µM) in mouse mast cells (c) or LAD2 cells (e). (*right*, *top*) Percentage of total cells activated by PACAP38 from mouse mast cells (*n* = 13 cultures) (c) or LAD2 cells (*n* = 12 cultures) (e). (*right*, *bottom*) Average Ca^2+^ transient trace of mouse mast cells (c) or LAD2 cells activated by PACAP38 (e). **d** Percentage of mast cells derived from wild type (WT) and MrgprB2-deficient (KO) mice activated by PACAP38 (10 µM) (*n* = 7 cultures). **f** Percentage of LAD2 cells activated by PACAP38 (1 µM) and treated with vehicle or QWF (*n* = 6 cultures). **g** (*left*) Representative confocal fluorescence images of avidin-stained (red) dura mater mast cells. (*right*) Percentage of degranulated mast cells (*n* = 4 mice per group). **h** (*left*) in vivo dura *Pirt*-GCaMP3 Ca^2+^ imaging in trigeminal nerve afferents with PACAP38 application. (*right*) Relative change of Ca^2+^ transient with PACAP38 (10 µM) through cranial window (*n* = 4 WT and 6 KO mice). **i** (*left*) Representative images showing in vivo fluorescence of dura blood vessels before and after PACAP38 (10 µM) application through cranial window. (*right*) Percentage change in blood vessel diameter after PACAP38 application (*n* = 4 WT and 6 KO mice). Error bars indicate S.E.M. *p < 0.05; **p < 0.01; ***p < 0.001; (d, f, h, i) two-tailed Student’s *t*-test, (g) one-way ANOVA with Tukey’s multiple comparison post-hoc test

Next, to investigate whether PACAP38 directly activates MrgprB2 and MRGPRX2 in vitro, we applied PACAP38 to HEK293 cells expressing mouse MrgprB2 or human MRGPRX2. PACAP38 application evoked Ca^2+^ transients in HEK293 cells expressing MrgprB2 or the human ortholog MRGPRX2 (Fig. 2a and b). PACAP38 application also evoked Ca^2+^ transients in isolated mouse mast cells and LAD2 human mast cells (Fig. 2c and e). PACAP38-induced Ca^2+^ transients were blocked by MrgprB2 deficiency in mouse cells and by QWF, a specific inhibitor of MRGPRX2, in human LAD2 cells (Fig. 2d and f). We asked whether PACAP38 elevation induces MrgprB2-dependent mast cell degranulation in vivo. To address this, we directly injected PACAP38 into dura mater. We collected dura mater 3 h after injection and counted degranulated mast cells. Degranulated mast cells were significantly increased in PACAP38-injected WT mice but not in MrgprB2-deficient mice (Fig. 2g). In addition, to confirm whether PACAP38 activates mast cells in dura mater in vivo, we used *Mrgprb2*-cre: GCaMP6 mice. In in vivo intact dura imaging, PACAP38 application significantly increased Ca^2+^ transients in dura mast cells (Supplementary Fig. 6, see Additional file 1), indicating that PACAP38 elevation in dura mater induces mast cell degranulation.

### PACAP38-MrgprB2 pathway activates the trigeminovascular unit

We examined whether the PACAP38-MrgprB2 pathway induces activation of the trigeminovascular unit in dura mater. The trigeminovascular unit, composed of trigeminal afferents and blood vessels, creates sensory signals of headache [40]. We first examined whether trigeminal afferents are activated by PACAP38 in vivo using *Pirt*-GCaMP3 Ca^2+^ imaging in intact dura mater. Direct PACAP38 application onto dura mater during in vivo imaging increased Ca^2+^ transients of dura trigeminal afferents (Fig. 2h). To test whether PACAP38 induces vasodilation, a primary event occurring during migraine headache [41], we assessed staining of blood vessels in dura mater with dextran-conjugated red-fluorescent dye. The diameter of blood vessels significantly increased after PACAP38 application (Fig. 2i). The increase in Ca^2+^ transients and vasodilation were dependent on MrgprB2 (Fig. 2h and i). Thus, it appears that elevation of PACAP38 in dura mater causes activation of trigeminal afferents and vasodilation through MrgprB2, which could be sufficient to sensitize trigeminal ganglion neurons.

### PACAP38-MrgprB2 pathway sensitizes TG neurons

**Fig. 3:**
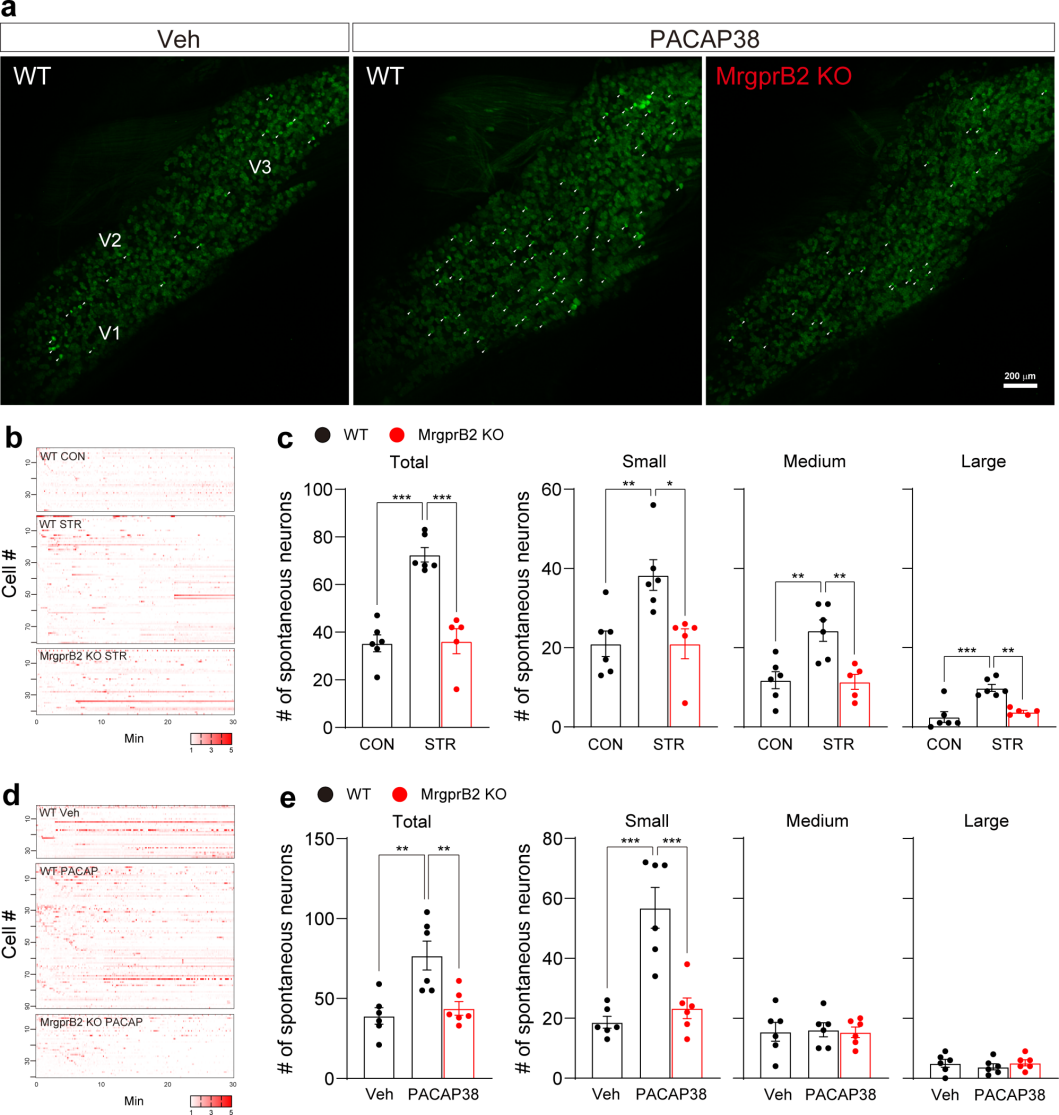
Activation of the PACAP38-MrgprB2 pathway increases the number of spontaneously activated TG neurons assessed by in vivo TG *Pirt*-GCaMP Ca^2+^ imaging. **a** Representative images of spontaneous in vivo activity assessed by TG *Pirt*-GCaMP3 Ca^2+^ imaging after PACAP38 dural injection. V1 (ophthalmic), V2 (maxillary), and V3 (mandibular) indicate the location of neuronal cell bodies in TG imaging. White arrowheads indicate Spontaneous Ca^2+^ activated neurons. **b and d** Representative heatmaps of spontaneously activated individual neurons in stressed (b) and PACAP38-injected (d) mice. **c and e** Number of total, small, medium, and large spontaneously activated neurons of each group in stressed (c) or PACAP38 injected (e) mice. (c) *n* = 6 WT CON, 6 WT STR, and 5 KO STR. (e) *n* = 6 mice per group. Small-diameter TG neurons (< 20 μm); medium (20–25 μm); large (> 25 μm). CON: control, KO: knock-out, STR: stress, Veh: vehicle, WT: wild-type. Error bars indicate S.E.M. **p < 0.01; ***p < 0.001; one-way ANOVA with Tukey’s multiple comparison post-hoc test

To assess spontaneous activities and sensitization in TG neurons of stressed mice, we monitored the activity of TG neurons in live animals using in vivo intact TG *Pirt*-GCaMP3 Ca^2+^ imaging the day after termination of repetitive stress. The total number of spontaneously activated neurons increased in stressed mice compared to control mice, and the increase was due to increases in all three subgroups, including small (< 20 μm)-, medium (20–25 μm)-, and large (> 25 μm)-diameter neurons (Fig. 3b and c). Stressed MrgprB2-deficient mice did not show increased spontaneous activities (Fig. 3b and c). We next asked whether elevated PACAP38 in dura mater mimics stress-induced spontaneous activities in TG neurons. To address this, we applied PACAP38 onto dura mater and monitored the activity of TG neurons 3 h after the application. Similar to the increase in spontaneously activated neurons seen in stressed WT but not MrgprB2-deficient animals following PACAP38 treatment (Fig. 3c), increased numbers of spontaneously activated neurons were seen in small-diameter neurons in WT but not in MrgprB2-deficient mice (Fig. 3d and e). Large and medium neurons did not differ from vehicle controls in either group of mice.

**Fig. 4:**
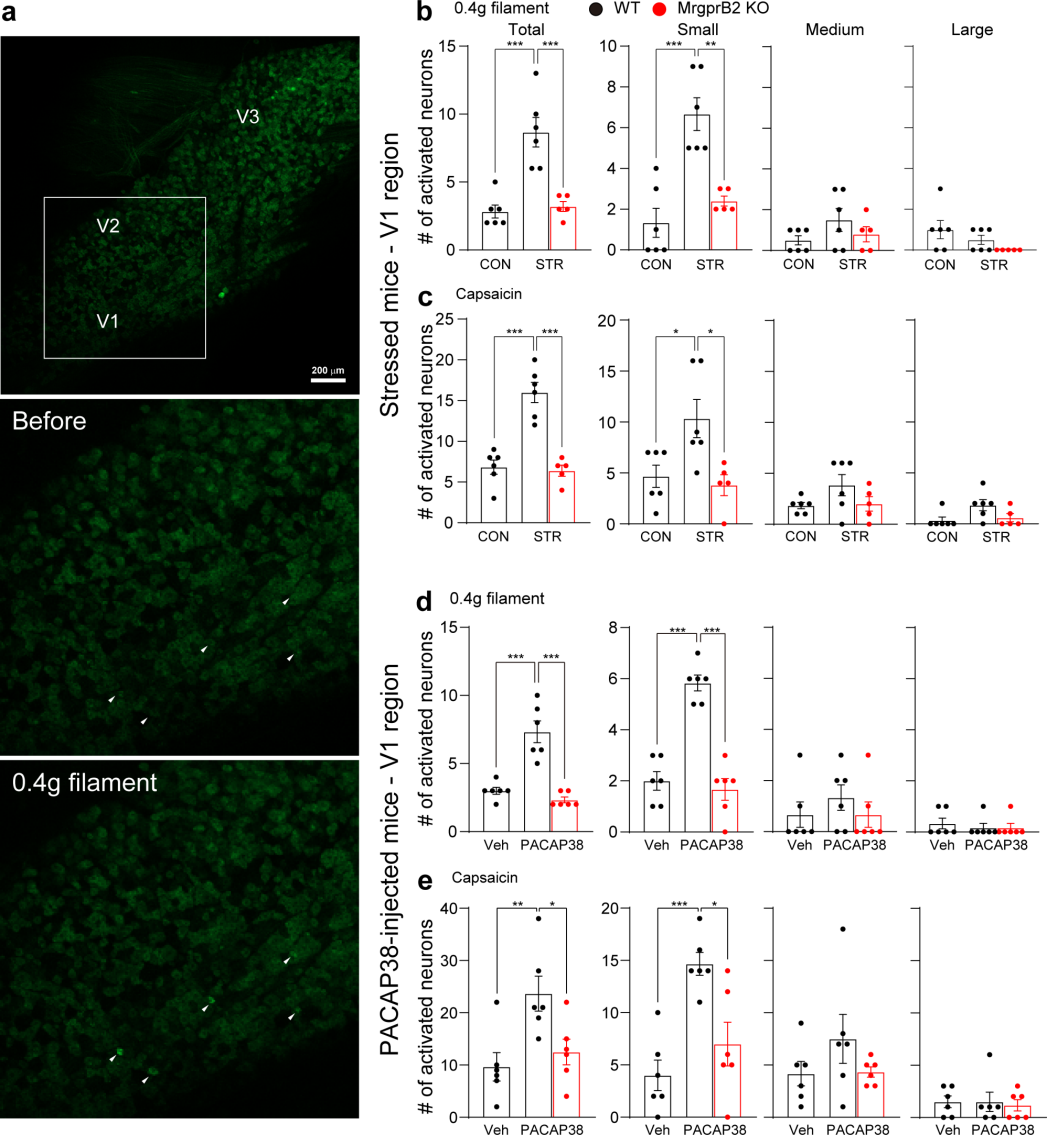
Activation of PACAP38-MrgprB2 pathway sensitizes TG neurons. **a** Representative images of in vivo TG neurons activated by 0.4 g von Frey filament application onto V1 region in PACAP38-injected mice. V1 (ophthalmic), V2 (maxillary), and V3 (mandibular) indicate the location of neuron cell bodies in TG imaging. White arrowheads indicate activated neurons. **b and c** Number of individual TG neurons activated by (b) 0.4 g von Frey filament or (c) capsaicin in stressed mice (*n* = 6 WT CON, 6 WT STR, and 5 KO STR). **d and e** Number of individual TG neurons activated by (d) 0.4 g von Frey filament or (e) capsaicin in PACAP38-injected mice (*n* = 6 mice per group). Small-diameter TG neurons (< 20 μm); medium (20–25 μm); large (> 25 μm). CON: control, KO: knock-out, STR: stress, Veh: vehicle, WT: wild-type. Error bars indicate S.E.M. *p < 0.05; **p < 0.01; ***p < 0.001; (b-e) one-way ANOVA with Tukey’s multiple comparison post-hoc test

Research on the pathophysiology of migraine headaches has revealed that cutaneous allodynia, a condition where pain is expressed in response to normally non-painful stimuli, can be observed when the periorbital and forehead skin areas are exposed to non-noxious stimulation during a headache episode [42, 43]. To check neuronal sensitization in TG, we applied von Frey filament (mechanical), hot/cold water (thermal), or capsaicin (chemical) to each orofacial region innervated by ophthalmic (V1) or maxillary (V2) TG neurons during in vivo intact TG *Pirt*-GCaMP3 Ca^2+^ imaging. Increased activation of TG neurons in stressed mice was seen following application of 0.4 g filament to the V1 region (Fig. 4b) but not following application to the V2 region (Supplementary Fig. 4a, see Additional file 1). This increase was due to increased activation of small-diameter neurons (Fig. 4b). Cold (4 °C) or hot (50 °C) water did not induce changes in either V1 or V2 regions of stressed mice (Supplementary Fig. 3a, b and 4b, c, see additional file 1). Capsaicin injection into the V1 region but not into the V2 region of stressed mice induced an increase in activated TG neurons (Fig. 4c and Supplementary Fig. 4d, see Additional file 1). PACAP38 injection into dura mater also increased the number of responding cells to von Frey filament and capsaicin injection in the V1 region (Fig. 4d and e) but not in the V2 region (Supplementary Fig. 5a and d, see Additional file 1), as was observed in stressed mice. These sensitizations were completely abolished in MrgprB2-deficient mice (Fig. 4d and e). No changes were induced in PACAP38-injected mice by thermal stimuli, including cold or hot water stimulation (Supplementary Fig. 3c, d and 5b, c, see Additional file 1). Capsaicin injection into the V1 region but not into the V2 region of PACAP38-injected mice also increased the number of activated TG neurons, similar to stressed mice (Fig. 4e, Supplementary Fig. 5d, see Additional file 1). These data indicate that PACAP38 elevation induced by stress in dura mater is sufficient to sensitize TG neurons via mast cell and MrgprB2.

### TNF-α receptor and TRPV1 contribute to stress-induced headache

**Fig. 5:**
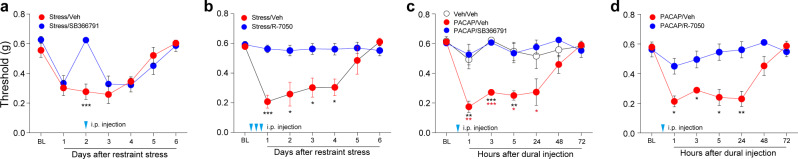
PACAP38 infusion and stress cause mechanical hypersensitivity in periorbital region via TNF-α receptor and TRPV1. **a-d** Head withdrawal thresholds tested by von Frey filament following restraint stress or PACAP38 dural injection. Mice were treated with i.p. injection of TRPV1-inhibitor (SB366791; 1 mg/kg) or TNF-α inhibitor (R-7050; 10 mg/kg) i.p. injection; or vehicle. (a) Withdrawal threshold in stressed mice treated with SB366791 (*n* = 6 mice per group). (b) Threshold in stressed mice treated with R-7050 (*n* = 5 mice per group). (c) Threshold in PACAP38-injected mice treated with SB366791 (*n* = 6 mice per group) *(black) Veh/Veh vs. PACAP38/Veh; *(red) PACAP38/Veh vs. PACAP38/SB366791. (d) Threshold in PACAP38-injected mice treated with R-7050 (*n* = 6 mice per group). Veh: vehicle. Error bars indicate S.E.M. *p < 0.05; **p < 0.01; ***p < 0.001; (a, b, d) two-tailed Student’s *t*-test, (c) two-way ANOVA with Tukey’s multiple comparison post-hoc test

Next, we asked how degranulated mast cells sensitize TG neurons. We previously confirmed that in alcohol withdrawal-induced headache mast cell degranulation mediated by MrgprB2 could induce headache behaviour via tumour necrosis factor-α (TNF-α) receptor and transient receptor potential vanilloid 1 (TRPV1) [44]. Since TRPV1 is mainly expressed in small-diameter neurons [45, 46], our observation of sensitization in small-diameter neurons (Figs. 3 and 4) led us to test whether TNF-α or/and TRPV1 contribute to stress-induced behaviours. Stress-induced hypersensitivity in the periorbital region was reversed by SB366791 (TRPV1 inhibitor) injection i.p. 1 h before facial von Frey testing (Fig. 5a). In addition, intraperitoneal treatment with R-7050 (TNF-α receptor inhibitor) prior to 3-day stress prevented stress-induced hypersensitivity (Fig. 5b). To confirm whether TRPV1 or/and TNF-α signalling acted downstream of PACAP38 elevation, we administered SB366791 and R-7050 intraperitoneally 1 h before PACAP38 injection into dura mater. We found that PACAP38-induced hypersensitivity in the periorbital region was also prevented by R-7050 and SB366791 injection (Fig. 5c and d). These results indicate that the PACAP38 pathway, including TNF-α and TRPV1, regulates stress-induced headache behaviour.

## Discussion

Although calcitonin gene-related peptide (CGRP)-based drugs exhibit some effectiveness, 40–50% of migraine patients do not respond to CGRP-based drugs [3, 47]. This limitation indicates that there is a need to develop new drugs that can complement CGRP-based drugs. PACAP38 has been studied as a new target molecule because of its vasodilation and headache-inducing ability [4]. Nevertheless, its mechanism of action remains to be determined. Unfortunately, a phase 2 randomized, double-blind, placebo-controlled study of AMG301, a monoclonal antibody to the PAC1 receptor, showed no benefit over placebo for migraine prevention [12]. Recently, Green and colleagues reported that the MrgprB2 axis mediates PACAP38-induced headache behaviours [48]. Here, we showed that PACAP38 directly activates MrgprB2 (mouse) and MRGPRX2 (human), and this PACAP38/-MrgprB2 pathway could sensitize TG neurons in stressed mice. These results provide evidence that PACAP38 causes headache via signalling through MrgprB2/MRGPRX2 of mast cells, not through conventional PACAP38 receptors, PAC1, VPAC1, and VPAC2. In addition, stress or/and PACAP38 elevation cause sensory sensitization via the TNF-α and TRPV1 pathway. These results are consistent with previous studies showing that degranulated mast cells release TNF-α which sensitizes nociceptors via TRPV1, resulting in pain [49, 50]. Indeed, migraineurs show elevated TNF-α levels in serum [51–53]. PACAP38 signalling, which is a CGRP independent pathway, mediates headache behaviour in a mouse migraine model [54]. Our results suggest that PACAP38-MrgprB2 could cause stress-induced headache behaviour via TNF-α and TRPV1 and highlight the the possibility that the PACAP38-MrgprB2 axis is a key mediator of stress-induced migraine, a promising avenue for the development of migraine treatments beyond CGRP-based therapies.

Mast cells are sensors as well as effectors and are first responders, acting as catalysts and recruiters to initiate, amplify, and prolong the activation of other immune and nervous system cells [55]. As effectors, mast cells can be degranulated, resulting in the release of various bioactive molecules [56]. Degranulation is a calcium-dependent process mediated by stimulated Ca^2+^ release from intracellular stores and Ca^2+^ entry through multiple channels at the plasma membrane [57]. Although MrgprB/X2 triggers mast cell degranulation via intracellular Ca^2+^ elevation, it remains unclear how MrgprB/X2 induces Ca^2+^ elevation. The cyclic adenosine monophosphate (cAMP) signalling pathway, a well-known downstream mediator of MrgprB/X2, indirectly triggers Ca^2+^ release from intracellular stores, which may not be sufficient to induce rapid MrgprB/X2-mediated Ca^2+^ elevation. Recent studies have shown that transient receptor potential canonical (TRPC) channels may contribute to MrgprB2-mediated mast cell activation independently of the FcεRI pathway [58, 59]. However, further mechanistic studies are required.

Among the bioactive molecules released by degranulated mast cells, TNF-a is attracting attention because of its pro-inflammatory capacity and the beneficial effects of TNF-a pathway antagonists on inflammation-related diseases [60, 61]. In contrast to the evidence implicating TNF-a in the pathogenesis of migraine [62–65] and our findings here, many people have received anti-TNFα treatment, and no change in migraine symptoms has been reported in these patients. This discrepancy may be due to the complexity of the cause of migraine in humans. There are multiple causes of migraine, and it is difficult to be certain that all causes are mediated by one mechanism. Here, we have shown that PACAP38-MrgprB2 induces stress-induced headache via TNF-a and TRPV1. However, a TRPV1 antagonist failed to show efficacy for the acute treatment of migraine in a phase 2 clinical trial [66]. Acute migraine is different from stress-induced migraine. Therefore, antagonists of TNF-a and TRPV1 may be effective in stress-related migraine. Research should be conducted to identify differences in each mechanism in different animal models such as nitroglycerin (NTG)-induced, sleep deprivation-induced, cortical spreading depression (CSD)-induced, and stress-induced headache.

Headache attacks in migraineurs are commonly triggered by stress [67, 68]. Stress, such as threatening situations, induces physiological responses. Activation of the hypothalamic-pituitary-adrenal (HPA) axis is a representative stress response that ultimately induces animals to act in response to environmental events [69]. Interestingly, hypothalamic activation occurs in the prodrome phase before a migraine attack [70]. Migraineurs also exhibit higher plasma levels of cortisol [43], a stress hormone controlled by the HPA axis, suggesting that hypothalamic activation involves the development of migraine headache. However, the bridge between the stress response and trigeminal activation is uncertain. Recently, it has been reported that neuronal activation in the hypothalamus regulates stress-induced headache behaviour, which is mediated by prolactin in female mice only [71, 72]. This mechanism is consistent with the notion that neuropeptides released by stress are capable of mediating stress-induced headache. Moreover, recently identified characteristics of MrgprB2 and MRGPRX2 provide new insight into the functional abilities of mast cells. MrgprB2 and MRGPRX2 can respond to various physiological peptides, including Substance P, oxytocin, somatostatin, cortistatin-14, dynorphin A, and PACAP [73], which allow mast cells to detect internal physiological changes. We show herein that the PACAP38-MrgprB2 pathway operates via sensitized TG neurons to mediate stress-induced headache behaviour in male mice. This finding suggests that MrgprB2 causes mast cells, which possess an ideal position adjacent to trigeminal afferents, to react to stress-induced homeostatic changes, resulting in headache behaviour. However, it remains to be determined whether the PACAP38-MrgprB2 headache behaviour-inducing pathway leads to headache behaviour in females.

Disruptions to homeostasis provoke adaptive restorative behaviours [74]. Given that stress is a potent disrupter of internal homeostasis [75], stress-induced headache may provide evolutionary benefits by encouraging sleeping or resting and limiting movement or activities as adaptive behaviours for rebalancing homeostasis [76]. The homeostatic afferent pathway starts with small-diameter primary sensory neurons sensing physiological changes, and encoded signals in sensory neurons are relayed to the central nervous system [77]. Consistent with this, we found that the PACAP38-MrgprB2 pathway induces sensitization in small-diameter TG neurons leading to headache behaviour, indicating that the PACAP38-MrgprB2 pathway mediates a signal between stress-induced unbalanced homeostasis and headache that may induce adaptive behaviour for rebalancing internal homeostasis. However, to fully unravel the unbalanced homeostasis and migraine headache, further research is crucial. Numerous peptides can interact with MrgprB2/MRGPRX2, and a variety of triggers can initiate headaches. It’s important to determine if common headache triggers like lack of sleep or skipping meals lead to changes in PACAP38 levels or affect other peptides that bind to MrgprB2. Additionally, the connection between these potential changes and headache symptoms needs to be examined. Nevertheless, our finding that activation of the PACAP38-MrgprB2/MRGPRX2 pathway causes headache behaviour suggests that migraine headache is a warning sign of stress-induced homeostatic imbalance.

## Conclusions

This study demonstrates that the PACAP38-MrgprB2 axis plays an important role in mediating stress-induced headache, revealing a novel therapeutic target for migraine treatment. By elucidating the role of this pathway, we identify a potential mechanism by which migraine serves as a warning of stress-induced homeostatic imbalances. This finding not only advances our understanding of migraine pathogenesis, but also opens avenues for the development of new treatments.

### Electronic supplementary material

Below is the link to the electronic supplementary material.

Supplementary Material 1

Supplementary Material 2

Supplementary Material 3

Supplementary Material 4

Supplementary Material 5

Supplementary Material 6

Supplementary Material 7

Supplementary Material 8

Supplementary Material 9

Supplementary Material 10

Supplementary Material 11

Supplementary Material 12

Supplementary Material 13

Supplementary Material 14

Supplementary Material 15

Supplementary Material 16

Supplementary Material 17

Supplementary Material 18

Supplementary Material 19

Supplementary Material 20

Supplementary Material 21

Supplementary Material 22

Supplementary Material 23

Supplementary Material 24

Supplementary Material 25

## Data Availability

No datasets were generated or analysed during the current study.

## Declarations

### Author details

^1^Department of Oral & Maxillofacial Surgery, School of Dentistry, TX, USA.

^2^Programs in Integrated Biomedical Sciences, Translational Sciences, Biomedical Engineering, Radiological Sciences, University of Texas Health Science Center at San Antonio, TX, USA.

### Supplemental information

Movie S1. Representative in vivo TG imaging of spontaneous activity from WT control mice, related to Fig. 3c.

Movie S2. Representative in vivo TG imaging of spontaneous activity from WT stressed mice, related to Fig. 3c.

Movie S3. Representative in vivo TG imaging of spontaneous activity from KO stressed mice, related to Fig. 3c.

Movie S4. Representative in vivo TG imaging of spontaneous activity from WT vehicle mice, related to Fig. 3e.

Movie S5. Representative in vivo TG imaging of spontaneous activity from WT PACAP38-injected mice, related to Fig. 3e.

Movie S6. Representative in vivo TG imaging of spontaneous activity from KO PACAP38-injected mice, related to Fig. 3e.

Movie S7. Representative in vivo TG imaging of activated neurons in response to 0.4 g filament stimulus from WT control mice, related to Fig. 4b.

Movie S8. Representative in vivo TG imaging of activated neurons in response to 0.4 g filament stimulus from WT stressed mice, related to Fig. 4b.

Movie S9. Representative in vivo TG imaging of activated neurons in response to 0.4 g filament stimulus from KO stressed mice, related to Fig. 4b.

Movie S10. Representative in vivo TG imaging of activated neurons in response to capsaicin from WT control mice, related to Fig. 4c.

Movie S11. Representative in vivo TG imaging of activated neurons in response to capsaicin from WT stressed mice, related to Fig. 4c.

Movie S12. Representative in vivo TG imaging of activated neurons in response to capsaicin from KO stressed mice, related to Fig. 4c.

Movie S13. Representative in vivo TG imaging of activated neurons in response to 0.4 g filament stimulus from WT vehicle mice, related to Fig. 4d.

Movie S14. Representative in vivo TG imaging of activated neurons in response to 0.4 g filament stimulus from WT PACAP38-injected mice, related to Fig. 4d.

Movie S15. Representative in vivo TG imaging of activated neurons in response to 0.4 g filament stimulus from KO PACAP38-injected mice, related to Fig. 4d.

Movie S16. Representative in vivo TG imaging of activated neurons in response to capsaicin from WT vehicle mice, related to Fig. 4e.

Movie S17. Representative in vivo TG imaging of activated neurons in response to capsaicin from WT PACAP38-injected mice, related to Fig. 4e.

Movie S18. Representative in vivo TG imaging of activated neurons in response to capsaicin from KO PACAP38-injected mice, related to Fig. 4e.
