# Supplementary figures and images for: PACAP38/mast-cell-specific receptor axis mediates repetitive stress-induced headache in mice
Source: J Headache Pain. 2024 May 28;25(1):87. doi: 10.1186/s10194-024-01786-3 (PMC11131290; doi:10.1186/s10194-024-01786-3)

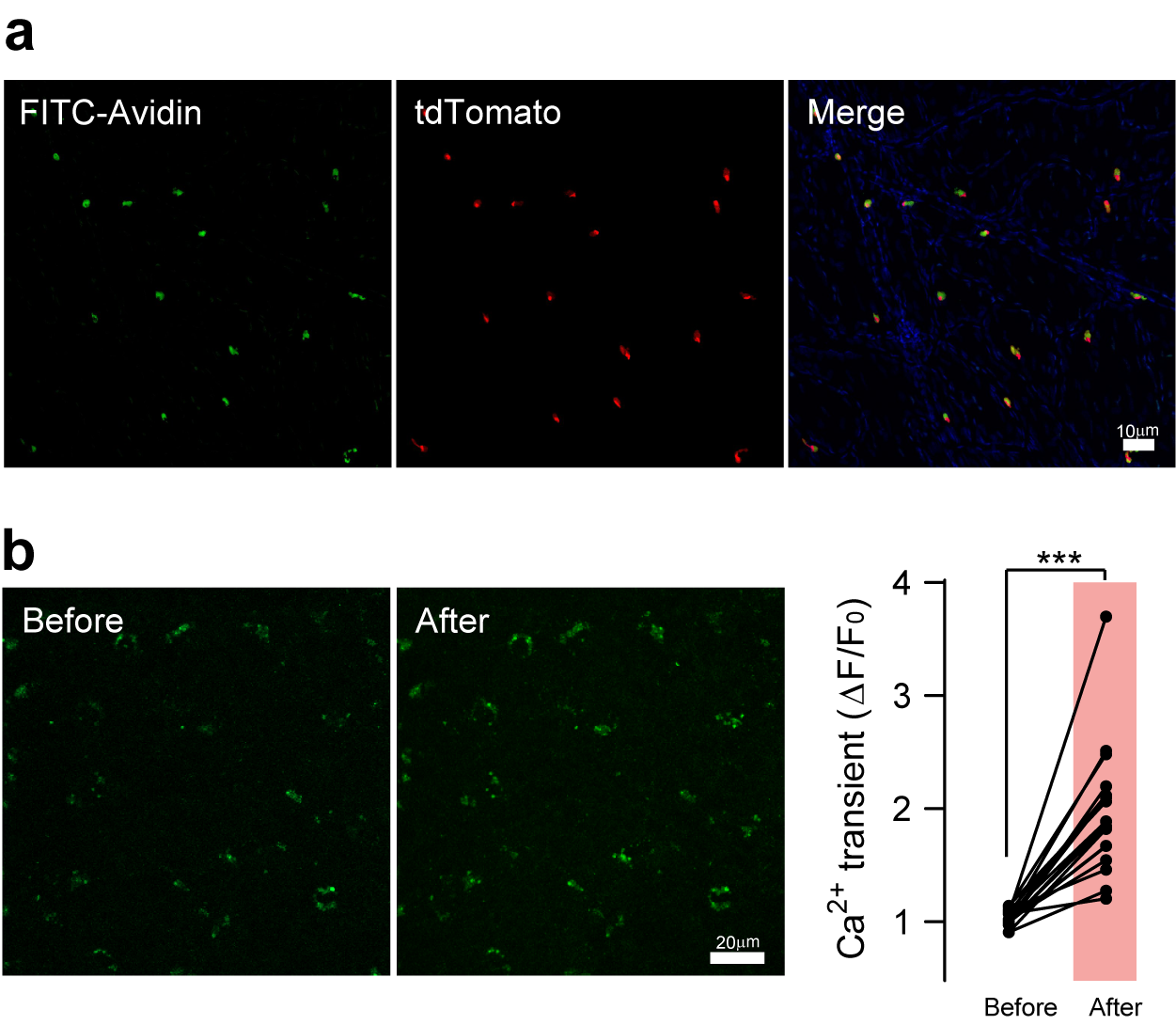

Supplement: Supplementary file 1 — Supplementary Material 1 [file 10194_2024_1786_MOESM1_ESM.tif]

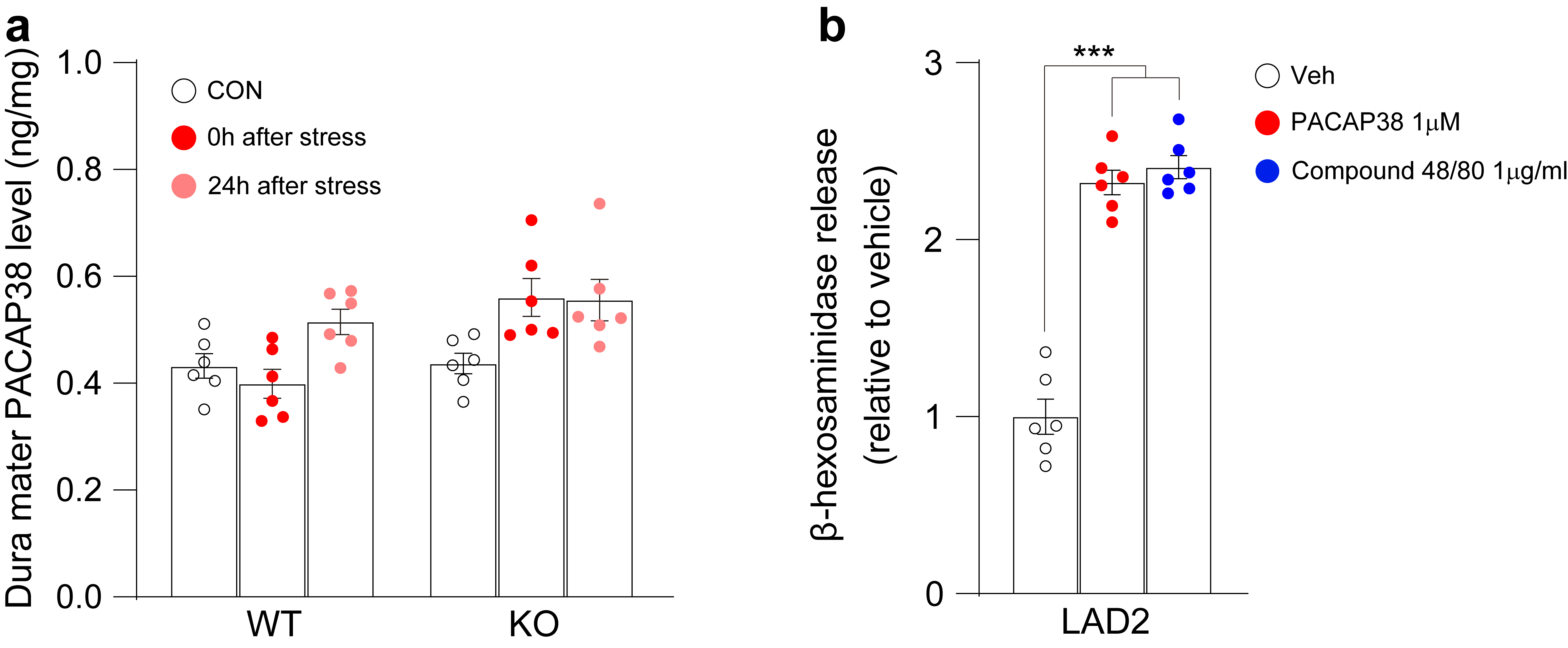

Supplement: Supplementary file 2 — Supplementary Material 2 [file 10194_2024_1786_MOESM2_ESM.tif]

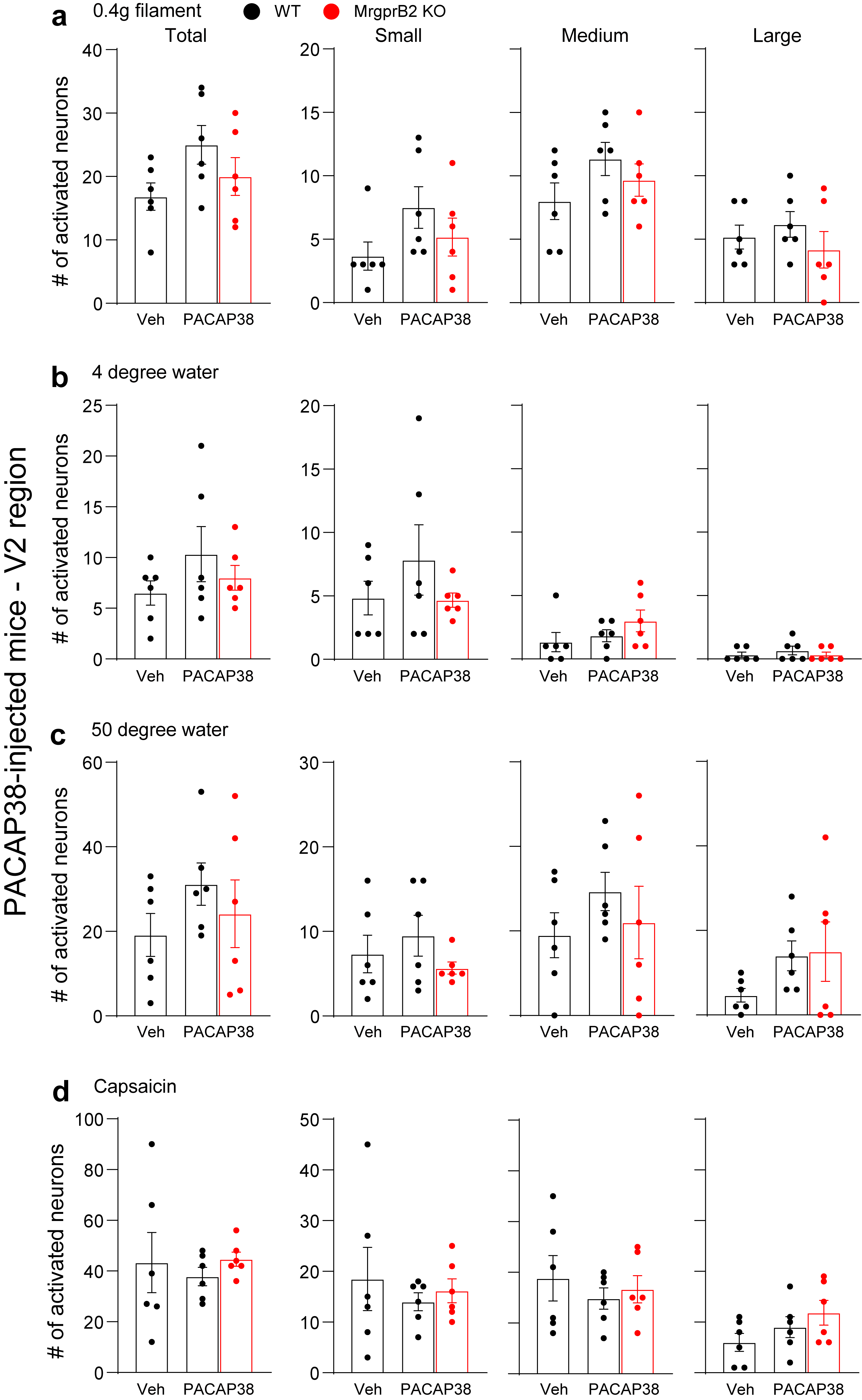

Supplement: Supplementary file 3 — Supplementary Material 3 [file 10194_2024_1786_MOESM3_ESM.tif]

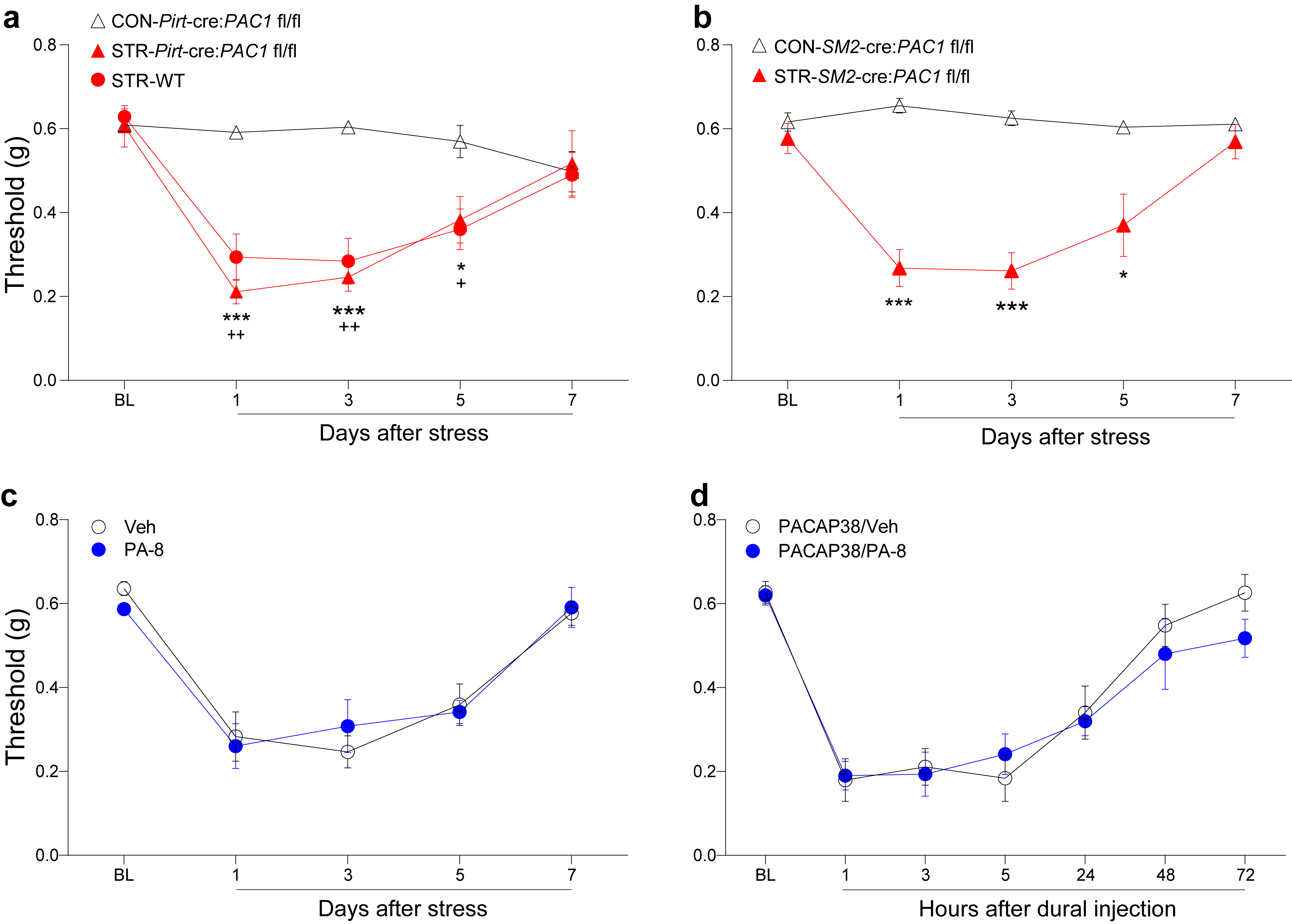

Supplement: Supplementary file 4 — Supplementary Material 4 [file 10194_2024_1786_MOESM4_ESM.tif]

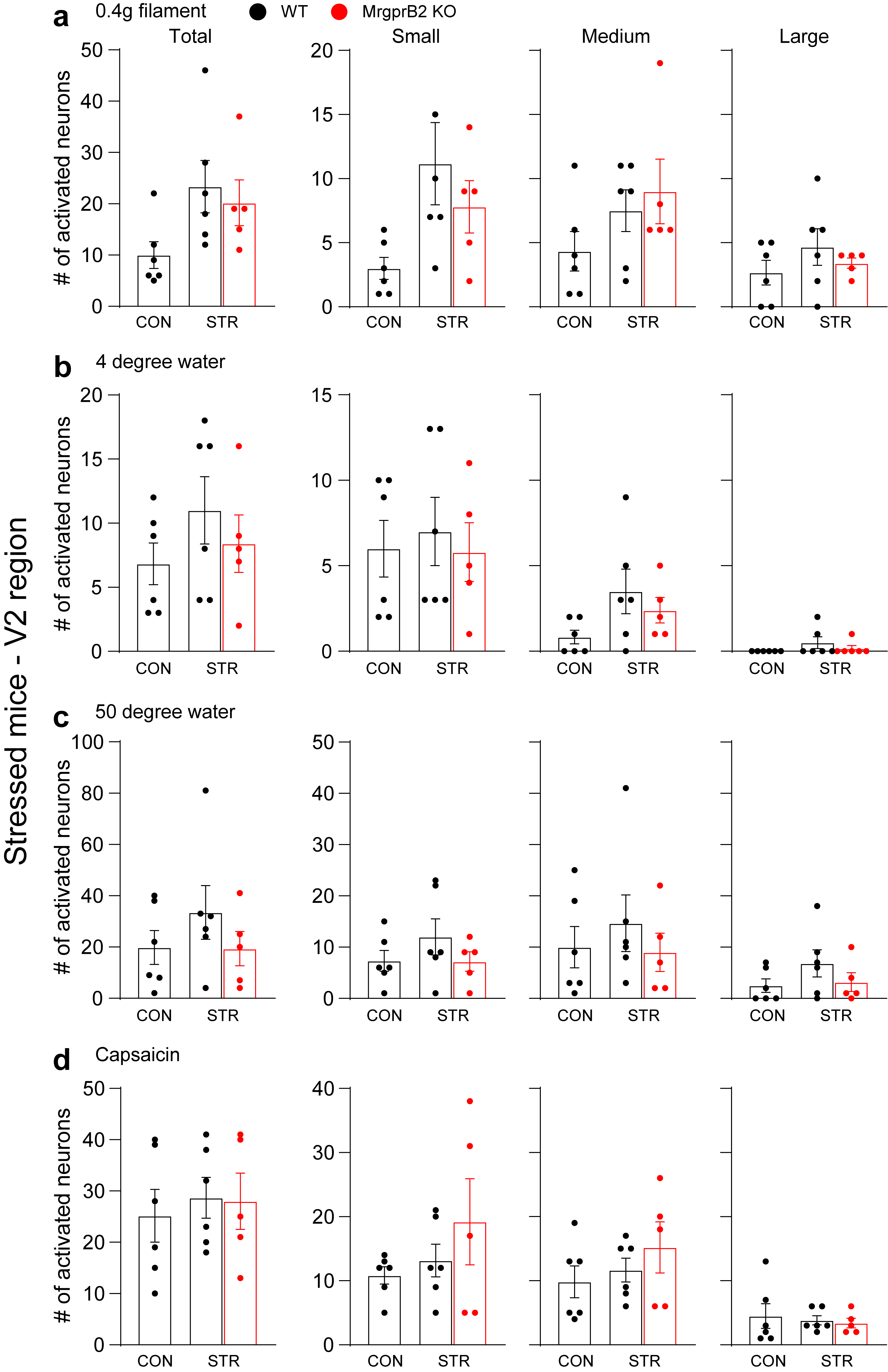

Supplement: Supplementary file 5 — Supplementary Material 5 [file 10194_2024_1786_MOESM5_ESM.tif]

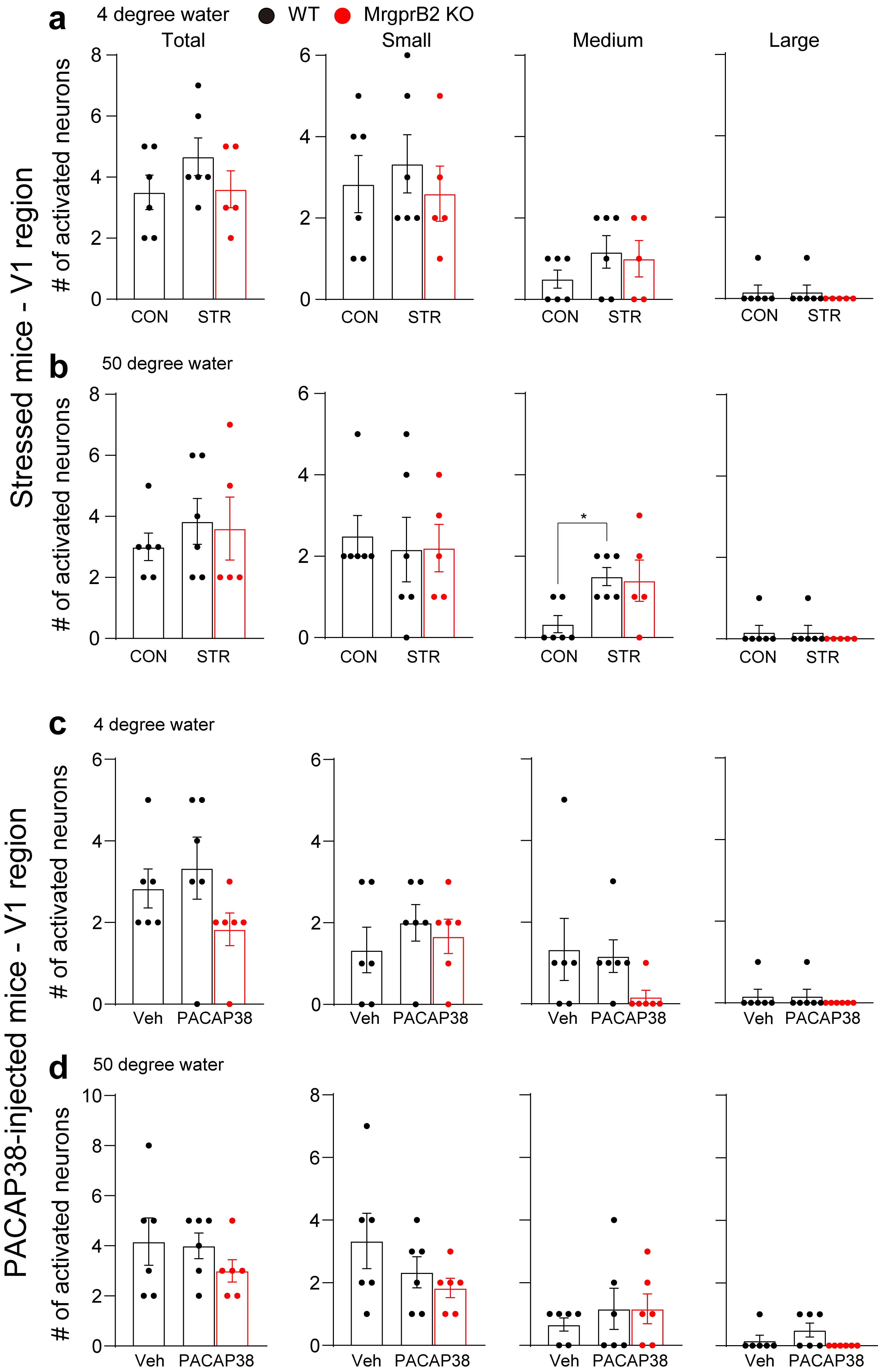

Supplement: Supplementary file 6 — Supplementary Material 6 [file 10194_2024_1786_MOESM6_ESM.tif]
