# Supplementary material for: PACAP38/mast-cell-specific receptor axis mediates repetitive stress-induced headache in mice
Source: J Headache Pain. 2024 May 28;25(1):87. doi: 10.1186/s10194-024-01786-3 (PMC11131290; doi:10.1186/s10194-024-01786-3)
Supplement: Supplementary file 25 — Supplementary Material 25 [file 10194_2024_1786_MOESM25_ESM.docx]

**Additional file 1**

Supplementary Figures


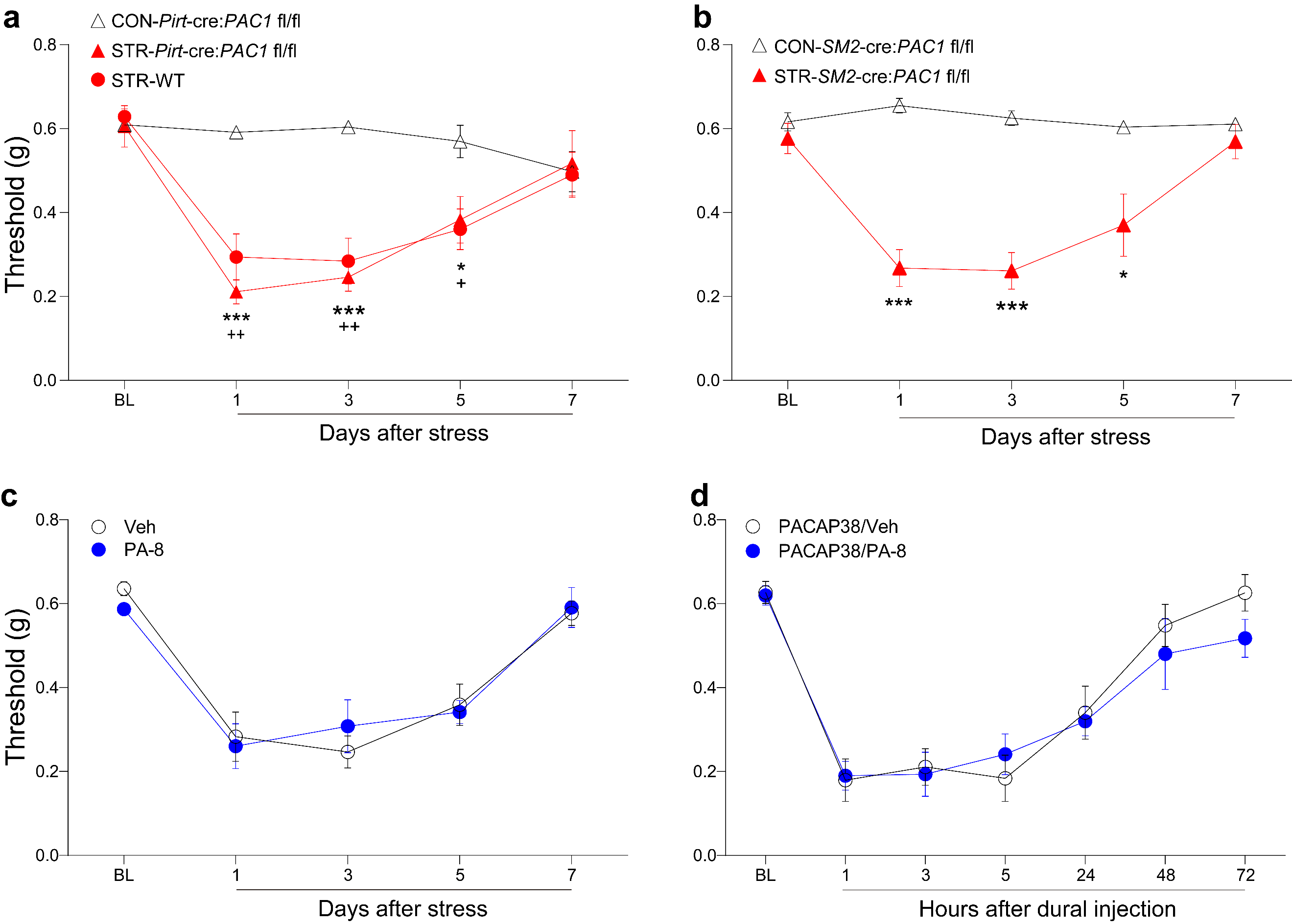


**Supplementary Figure 1.** PAC1 is not involved in repetitive stress or PACAP38 dural injection-induced headache behavior. **(a and b)** Facial mechanical withdrawal thresholds tested by von Frey filament after restraint stress; (a) *Pirt*-cre:*PAC1* floxed/floxed mice (n = 6 mice per group), *CON-*Pirt*-cre:*PAC1* fl/fl vs. STR-*Pirt*-cre:*PAC1* fl/fl; +STR-WT vs. STR-*Pirt*-cre:*PAC1* fl/fl; (b) *SM2*-cre-ERT2:*PAC1* floxed/floxed mice (n = 6 mice per group), STR-*SM2*-cre:*PAC1 fl/fl vs*. CON-*SM2*-cre:*PAC1 fl/fl.* **(c and d)** Facial mechanical withdrawal thresholds tested by von Frey filament after restraint stress (C, n = 7-8 mice per group) and PACAP38 dural injection (D, n = 6 mice per group) with PA-8, an inhibitor of PAC1, (10 mg/kg) i.p. injection. CON: control, STR: stress, Veh: vehicle, KO: knockout, WT: wild-type. Error bars indicate S.E.M. **p* < 0.05; ***p* < 0.01; ****p* < 0.001, (a) two-way ANOVA with Tukey’s multiple comparison post-hoc test; (b) two-tailed Student’s *t*-test.


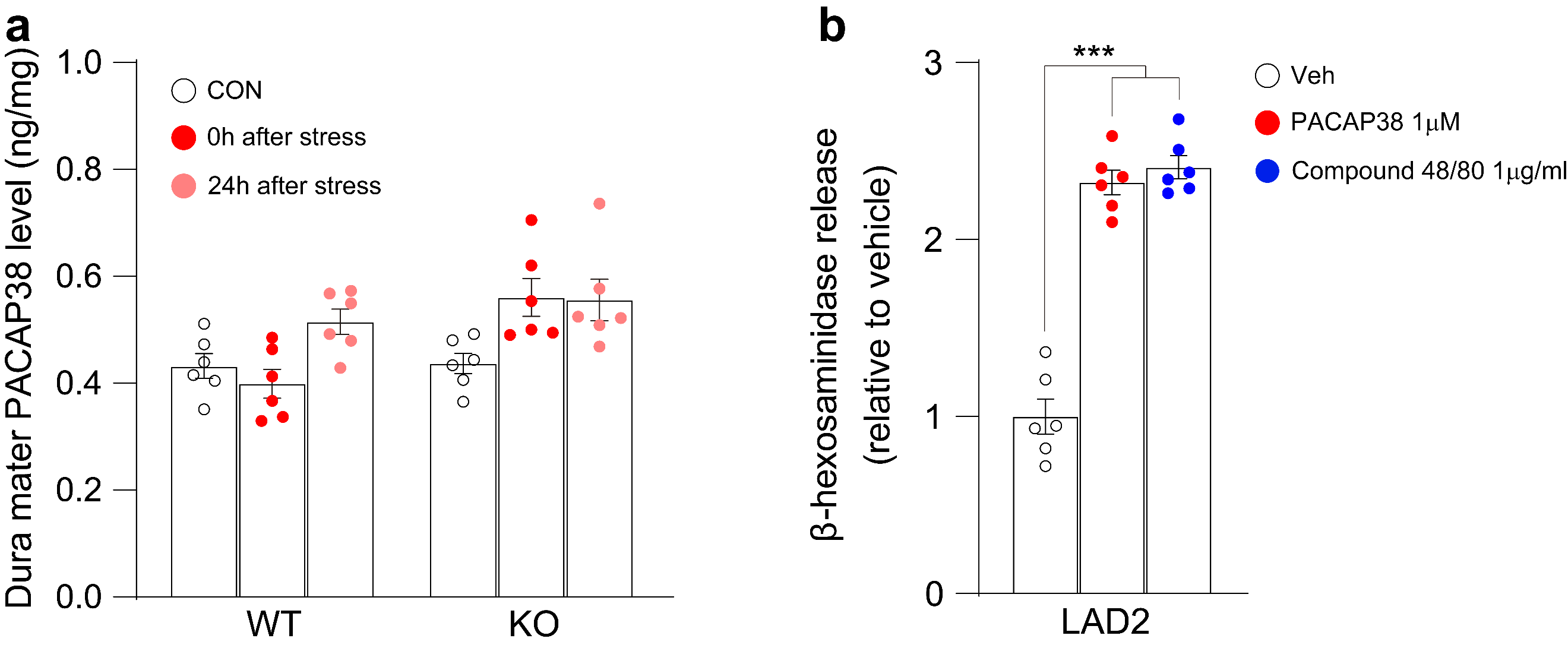


**Supplementary Figure 2.** Effects of repetitive stress on PACAP38 levels of dura mater and impact of PACAP38 on degranulation in LAD2 mast cells. **(a)** PACAP38 concentration measured in dura mater (n = 6 mice per group). **(b)** Quantification of β-hexosaminidase release from human LAD2 mast cells (n = 6 wells per group). Veh: vehicle. Error bars indicate S.E.M. ****p* < 0.001, one-way ANOVA with Tukey’s multiple comparison post-hoc test.


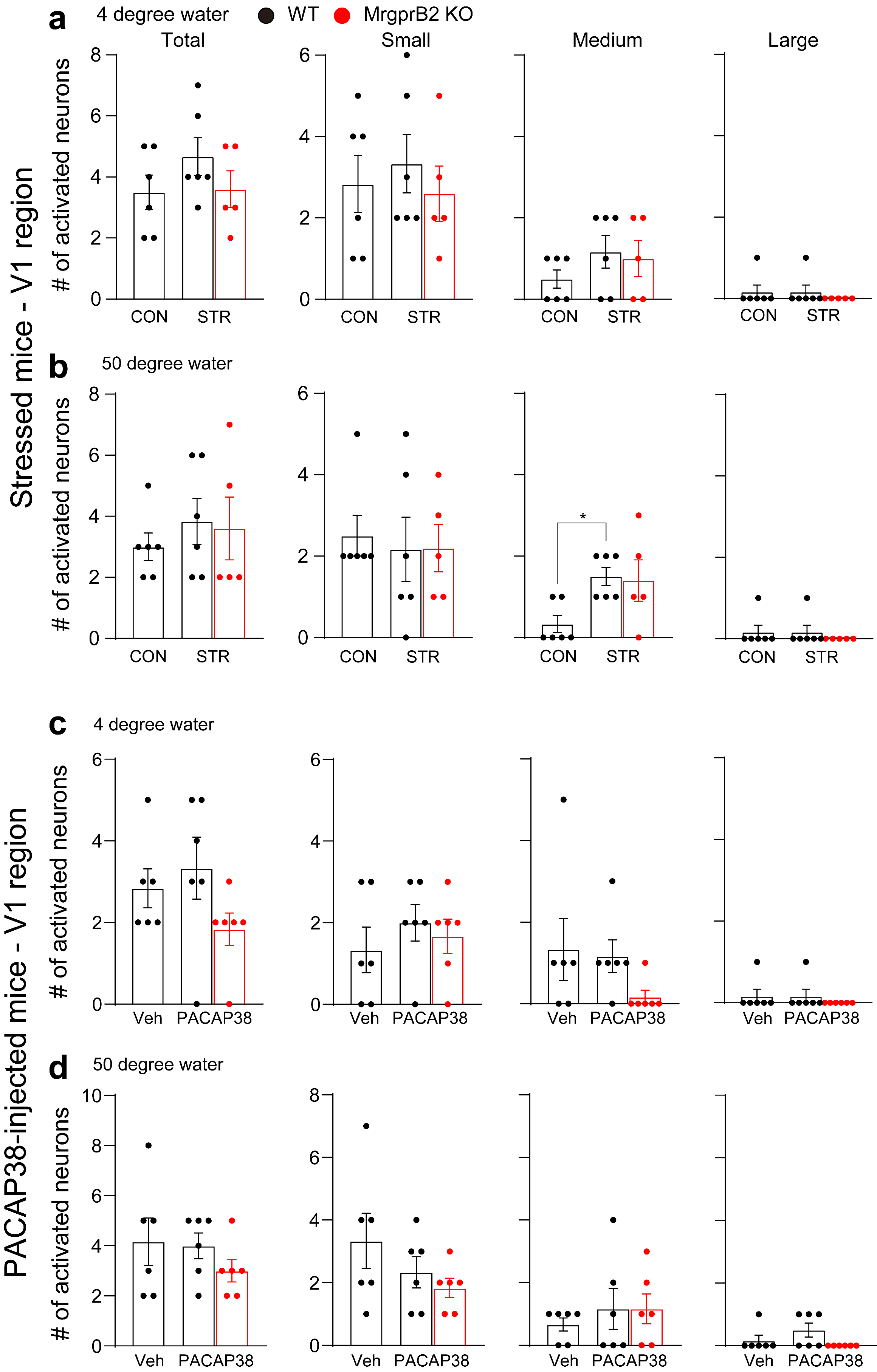


**Supplementary Figure 3.** Activated neurons responding to cold or hot water in V1 region of stressed or PACAP38-injected mice. **(a and b)** Number of activated TG neurons responding to cold (a) or hot (b) water in stressed mice (n = 6 WT CON, 6 WT STR, and 5 KO STR). **(c and d)** Number of activated TG neurons responding to cold (c) or hot (d) water in PACAP38-injected mice (n = 6 mice per group). Small-diameter TG neurons (<20 µm); medium (20-25 µm); large (>25 µm). CON: control, STR: stress, Veh: vehicle. Error bars indicate S.E.M. **p* < 0.05 by one-way ANOVA with Tukey’s multiple comparison post-hoc test.

**
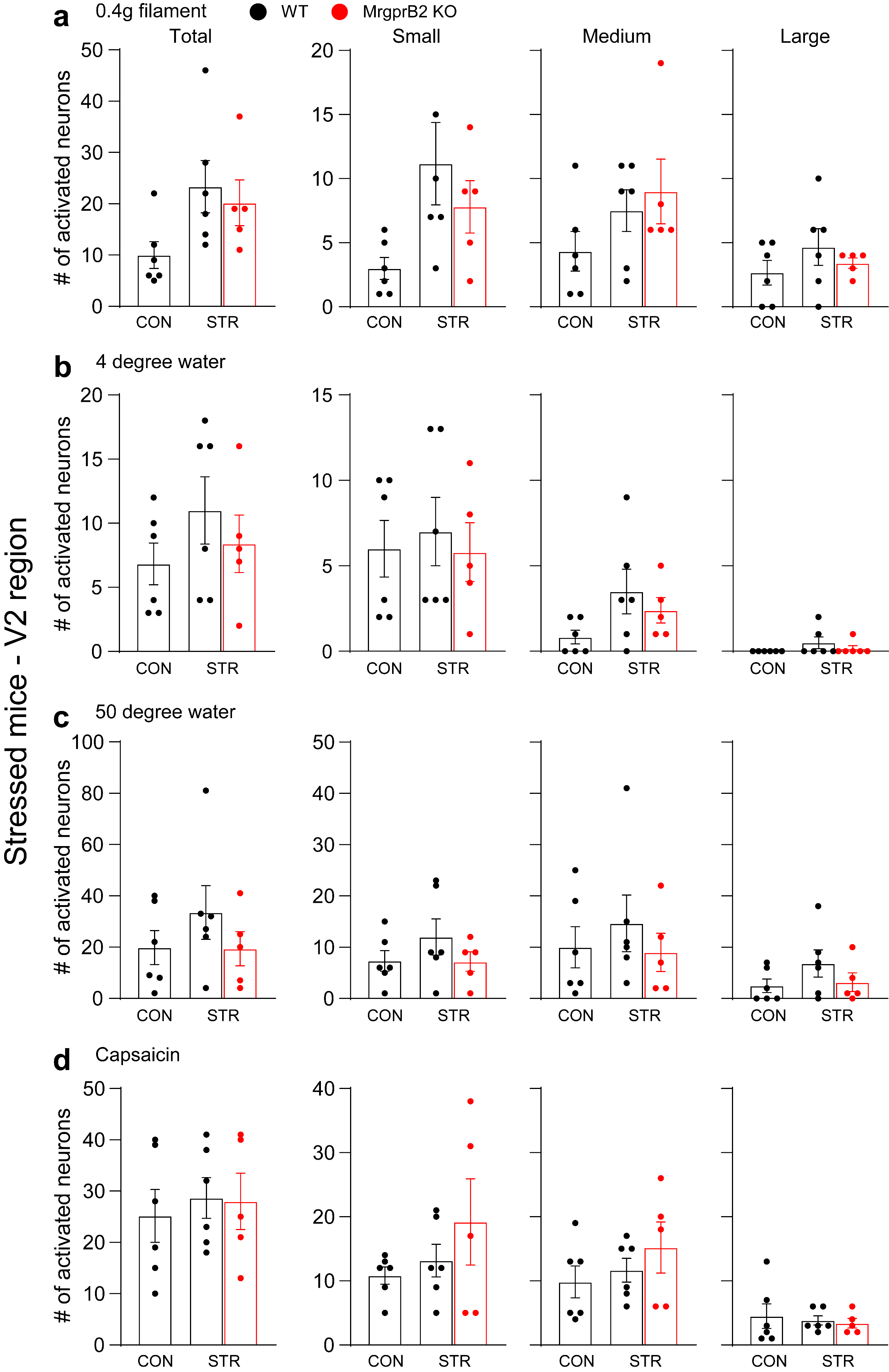
**

**Supplementary Figure 4.** Activated neurons responding to various stimuli in V2 region of stressed mice. **(a-d)** Number of TG neurons activated by 0.4 g von Frey filament (a), cold water (b), hot water (c), capsaicin (d). For each condition: n = 6 WT CON, 6 WT STR, and 5 KO STR. Small-diameter neurons (<20 µm); medium (20-25 µm); large (>25 µm). CON: control, STR: stress. Error bars indicate S.E.M.


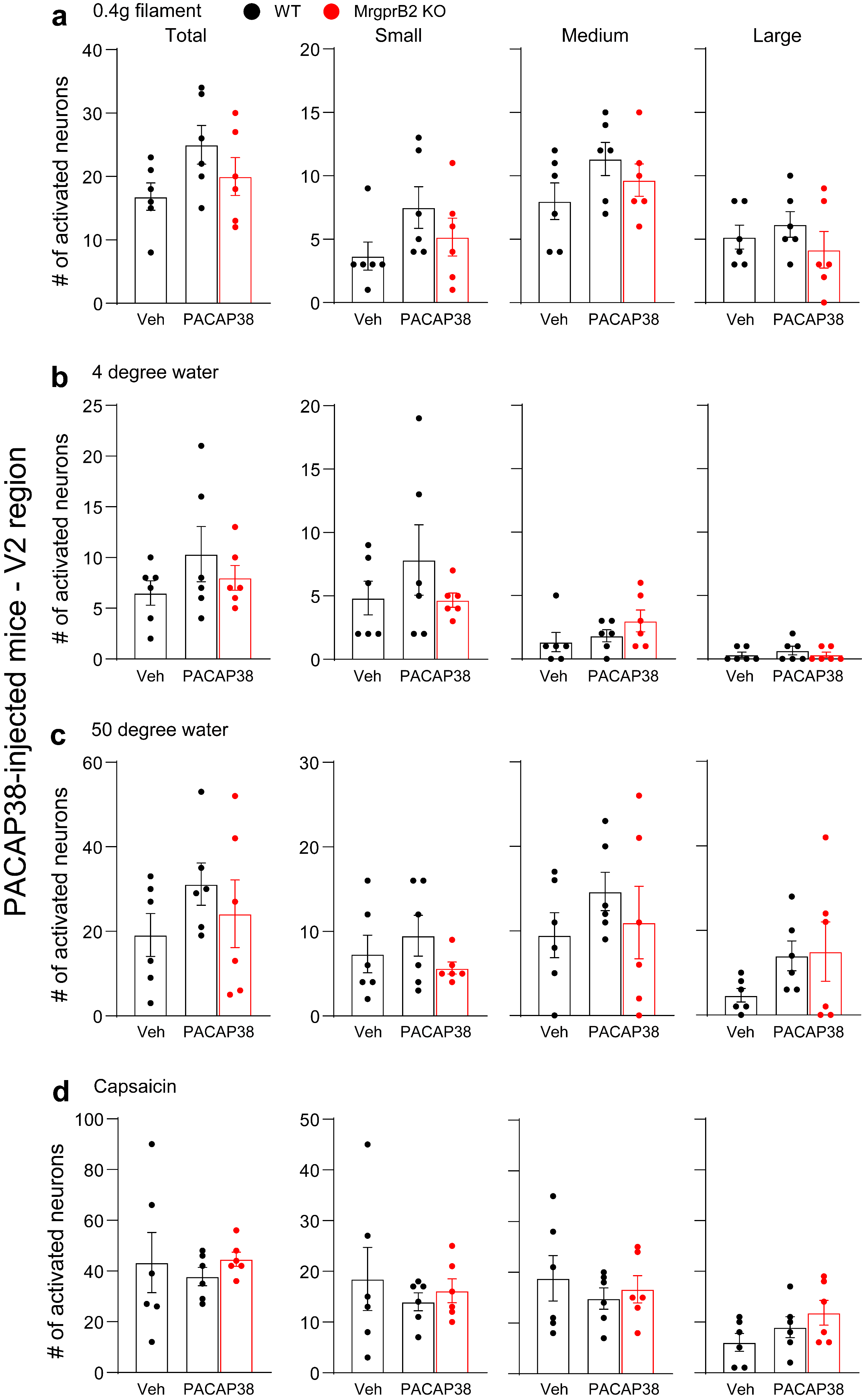


**Supplementary Figure 5.** Activated neurons responding to various stimuli in V2 region of PACAP38-injected mice. **(a-d)** Number of TG neurons activated by 0.4 g von Frey filament (a), cold water (b), hot water (c), or capsaicin (d) (n = 6 mice per group). Small-diameter neurons (<20 µm); medium (20-25 µm); large (>25 µm). Veh: vehicle. Error bars indicate S.E.M.


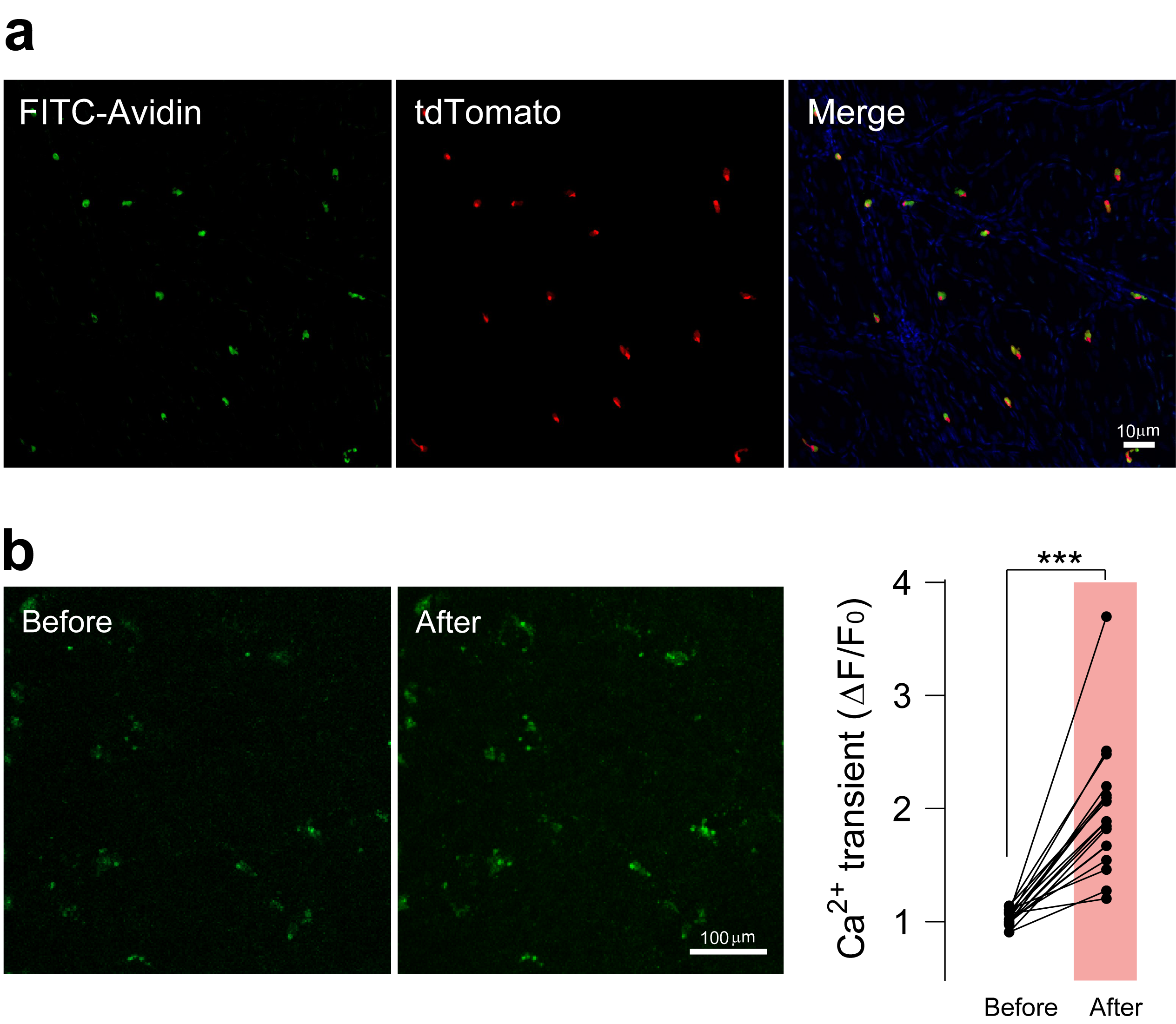


**Supplementary Figure 6.** Effect of PACAP38 on Dura Mater Mast Cells assessed by *in vivo* Dura Mater GCaMP3 Imaging. **(a)** Representative confocal images from *MrgprB2*-cre:tdTomato mice. *MrgprbB2*-cre mice were mated to tdTomato reporter mice, and tdTomato (red) expression was colocalized with avidin staining (green), a marker of mast cells. **(b)** (*left*) Representative Ca^2+^ images of *in vivo* intact dura mast cells (*MrgprB2*-cre-GCaMP6) before and after PACAP38 application. (*right*) Relative changes in Ca^2+^ transients with PACAP38 (10 µM) application through cranial window (n = 18 cells). Error bars indicate S.E.M. ****p* < 0.001, two-tailed Student’s *t*-test.
